# Supplementary material for: A longitudinal study of four-year changes in physical fitness among university students before and after COVID-19: 2019–2022
Source: PLoS One. 2025 Oct 10;20(10):e0334088. doi: 10.1371/journal.pone.0334088 (PMC12513638; doi:10.1371/journal.pone.0334088)
Supplement: S1 File — (DOCX) [file pone.0334088.s001.docx]

***Supplementary Material***

| **One-way ANOVA results （male）** | | | | | | |
| --- | --- | --- | --- | --- | --- | --- |
|  | | square sum | df | mean square | F | p |
| height | Intergroup | 199.721 | 3 | 66.574 | 1.745 | .156 |
|  | Within Groups | 183933.685 | 4820 | 38.161 |  |  |
|  | Total | 184133.406 | 4823 |  |  |  |
| weight | Intergroup | 9342.979 | 3 | 3114.326 | 19.698 | .000 |
|  | Within Groups | 762041.658 | 4820 | 158.100 |  |  |
|  | Total | 771384.637 | 4823 |  |  |  |
| BMI | Intergroup | 869.047 | 3 | 289.682 | 20.095 | .000 |
|  | Within Groups | 69483.346 | 4820 | 14.416 |  |  |
|  | Total | 70352.394 | 4823 |  |  |  |
| Vital capacity | Intergroup | 71423779.311 | 3 | 23807926.437 | 132.750 | .000 |
|  | Within Groups | 864441189.673 | 4820 | 179344.645 |  |  |
|  | Total | 935864968.984 | 4823 |  |  |  |
| 50-m run | Intergroup | .456 | 3 | .152 | .690 | .558 |
|  | Within Groups | 1061.676 | 4820 | .220 |  |  |
|  | Total | 1062.132 | 4823 |  |  |  |
| long-jump | Intergroup | 107613.269 | 3 | 35871.090 | 128.238 | .000 |
|  | Within Groups | 1348265.269 | 4820 | 279.723 |  |  |
|  | Total | 1455878.537 | 4823 |  |  |  |
| sit-reach | Intergroup | 1519.357 | 3 | 506.452 | 11.032 | .000 |
|  | Within Groups | 221282.892 | 4820 | 45.909 |  |  |
|  | Total | 222802.250 | 4823 |  |  |  |
| 1,000 m run | Intergroup | 317578.948 | 3 | 105859.649 | 246.005 | .000 |
|  | Within Groups | 2074120.096 | 4820 | 430.315 |  |  |
|  | Total | 2391699.044 | 4823 |  |  |  |
| pull-ups | Intergroup | 6.157 | 3 | 2.052 | .170 | .916 |
|  | Within Groups | 58090.002 | 4820 | 12.052 |  |  |
|  | Total | 58096.158 | 4823 |  |  |  |

Notes: ANOVA:Analysis of Variance

| **Multiple comparisons (LSD) Male** | | | | | | | |
| --- | --- | --- | --- | --- | --- | --- | --- |
| Dependent Variable | (I) year | (J) year | Mean Deviation (I-J) | standard error | significance | 95% CI | |
|  |  |  |  |  |  | lower-bound | upper-bound |
| height | 2019 | 2020 | .000 | .252 | 1.000 | -.49 | .49 |
|  |  | 2021 | -.424 | .252 | .092 | -.92 | .07 |
|  |  | 2022 | -.388 | .252 | .123 | -.88 | .11 |
|  | 2020 | 2019 | .000 | .252 | 1.000 | -.49 | .49 |
|  |  | 2021 | -.424 | .252 | .092 | -.92 | .07 |
|  |  | 2022 | -.388 | .252 | .123 | -.88 | .11 |
|  | 2021 | 2019 | .424 | .252 | .092 | -.07 | .92 |
|  |  | 2020 | .424 | .252 | .092 | -.07 | .92 |
|  |  | 2022 | .036 | .252 | .885 | -.46 | .53 |
|  | 2022 | 2019 | .388 | .252 | .123 | -.11 | .88 |
|  |  | 2020 | .388 | .252 | .123 | -.11 | .88 |
|  |  | 2021 | -.036 | .252 | .885 | -.53 | .46 |
| weight | 2019 | 2020 | .000 | .512 | 1.000 | -1.00 | 1.00 |
|  |  | 2021 | -1.163^*^ | .512 | .023 | -2.17 | -.16 |
|  |  | 2022 | -3.409^*^ | .512 | .000 | -4.41 | -2.40 |
|  | 2020 | 2019 | .000 | .512 | 1.000 | -1.00 | 1.00 |
|  |  | 2021 | -1.163^*^ | .512 | .023 | -2.17 | -.16 |
|  |  | 2022 | -3.409^*^ | .512 | .000 | -4.41 | -2.40 |
|  | 2021 | 2019 | 1.163^*^ | .512 | .023 | .16 | 2.17 |
|  |  | 2020 | 1.163^*^ | .512 | .023 | .16 | 2.17 |
|  |  | 2022 | -2.246^*^ | .512 | .000 | -3.25 | -1.24 |
|  | 2022 | 2019 | 3.409^*^ | .512 | .000 | 2.40 | 4.41 |
|  |  | 2020 | 3.409^*^ | .512 | .000 | 2.40 | 4.41 |
|  |  | 2021 | 2.246^*^ | .512 | .000 | 1.24 | 3.25 |
| BMI | 2019 | 2020 | .000 | .155 | 1.000 | -.30 | .30 |
|  |  | 2021 | -.271 | .155 | .080 | -.57 | .03 |
|  |  | 2022 | -1.037^*^ | .155 | .000 | -1.34 | -.73 |
|  | 2020 | 2019 | .000 | .155 | 1.000 | -.30 | .30 |
|  |  | 2021 | -.271 | .155 | .080 | -.57 | .03 |
|  |  | 2022 | -1.037^*^ | .155 | .000 | -1.34 | -.73 |
|  | 2021 | 2019 | .271 | .155 | .080 | -.03 | .57 |
|  |  | 2020 | .271 | .155 | .080 | -.03 | .57 |
|  |  | 2022 | -.766^*^ | .155 | .000 | -1.07 | -.46 |
|  | 2022 | 2019 | 1.037^*^ | .155 | .000 | .73 | 1.34 |
|  |  | 2020 | 1.037^*^ | .155 | .000 | .73 | 1.34 |
|  |  | 2021 | .766^*^ | .155 | .000 | .46 | 1.07 |
| Vital capacity | 2019 | 2020 | 101.585^*^ | 17.246 | .000 | 67.77 | 135.39 |
|  |  | 2021 | -233.789^*^ | 17.246 | .000 | -267.60 | -199.98 |
|  |  | 2022 | -53.944^*^ | 17.246 | .002 | -87.75 | -20.13 |
|  | 2020 | 2019 | -101.585^*^ | 17.246 | .000 | -135.39 | -67.77 |
|  |  | 2021 | -335.373^*^ | 17.246 | .000 | -369.18 | -301.56 |
|  |  | 2022 | -155.528^*^ | 17.246 | .000 | -189.34 | -121.72 |
|  | 2021 | 2019 | 233.789^*^ | 17.246 | .000 | 199.98 | 267.60 |
|  |  | 2020 | 335.373^*^ | 17.246 | .000 | 301.56 | 369.18 |
|  |  | 2022 | 179.845^*^ | 17.246 | .000 | 146.04 | 213.65 |
|  | 2022 | 2019 | 53.944^*^ | 17.246 | .002 | 20.13 | 87.75 |
|  |  | 2020 | 155.528^*^ | 17.246 | .000 | 121.72 | 189.34 |
|  |  | 2021 | -179.845^*^ | 17.246 | .000 | -213.65 | -146.04 |
| 50-m run | 2019 | 2020 | .000 | .019 | 1.000 | -.04 | .04 |
|  |  | 2021 | -.020 | .019 | .304 | -.06 | .02 |
|  |  | 2022 | -.019 | .019 | .314 | -.06 | .02 |
|  | 2020 | 2019 | .000 | .019 | 1.000 | -.04 | .04 |
|  |  | 2021 | -.020 | .019 | .304 | -.06 | .02 |
|  |  | 2022 | -.019 | .019 | .314 | -.06 | .02 |
|  | 2021 | 2019 | .020 | .019 | .304 | -.02 | .06 |
|  |  | 2020 | .020 | .019 | .304 | -.02 | .06 |
|  |  | 2022 | .000 | .019 | .983 | -.04 | .04 |
|  | 2022 | 2019 | .019 | .019 | .314 | -.02 | .06 |
|  |  | 2020 | .019 | .019 | .314 | -.02 | .06 |
|  |  | 2021 | .000 | .019 | .983 | -.04 | .04 |
| long-jump | 2019 | 2020 | 10.759^*^ | .681 | .000 | 9.42 | 12.09 |
|  |  | 2021 | -.254 | .681 | .710 | -1.59 | 1.08 |
|  |  | 2022 | 7.190^*^ | .681 | .000 | 5.85 | 8.53 |
|  | 2020 | 2019 | -10.759^*^ | .681 | .000 | -12.09 | -9.42 |
|  |  | 2021 | -11.012^*^ | .681 | .000 | -12.35 | -9.68 |
|  |  | 2022 | -3.569^*^ | .681 | .000 | -4.90 | -2.23 |
|  | 2021 | 2019 | .254 | .681 | .710 | -1.08 | 1.59 |
|  |  | 2020 | 11.012^*^ | .681 | .000 | 9.68 | 12.35 |
|  |  | 2022 | 7.444^*^ | .681 | .000 | 6.11 | 8.78 |
|  | 2022 | 2019 | -7.190^*^ | .681 | .000 | -8.53 | -5.85 |
|  |  | 2020 | 3.569^*^ | .681 | .000 | 2.23 | 4.90 |
|  |  | 2021 | -7.444^*^ | .681 | .000 | -8.78 | -6.11 |
| sit-reach | 2019 | 2020 | .000 | .276 | 1.000 | -.54 | .54 |
|  |  | 2021 | .579^*^ | .276 | .036 | .04 | 1.12 |
|  |  | 2022 | 1.368^*^ | .276 | .000 | .83 | 1.91 |
|  | 2020 | 2019 | .000 | .276 | 1.000 | -.54 | .54 |
|  |  | 2021 | .579^*^ | .276 | .036 | .04 | 1.12 |
|  |  | 2022 | 1.368^*^ | .276 | .000 | .83 | 1.91 |
|  | 2021 | 2019 | -.579^*^ | .276 | .036 | -1.12 | -.04 |
|  |  | 2020 | -.579^*^ | .276 | .036 | -1.12 | -.04 |
|  |  | 2022 | .789^*^ | .276 | .004 | .25 | 1.33 |
|  | 2022 | 2019 | -1.368^*^ | .276 | .000 | -1.91 | -.83 |
|  |  | 2020 | -1.368^*^ | .276 | .000 | -1.91 | -.83 |
|  |  | 2021 | -.789^*^ | .276 | .004 | -1.33 | -.25 |
| 1,000 m run | 2019 | 2020 | .000 | .845 | 1.000 | -1.66 | 1.66 |
|  |  | 2021 | -16.363^*^ | .845 | .000 | -18.02 | -14.71 |
|  |  | 2022 | -16.090^*^ | .845 | .000 | -17.75 | -14.43 |
|  | 2020 | 2019 | .000 | .845 | 1.000 | -1.66 | 1.66 |
|  |  | 2021 | -16.363^*^ | .845 | .000 | -18.02 | -14.71 |
|  |  | 2022 | -16.090^*^ | .845 | .000 | -17.75 | -14.43 |
|  | 2021 | 2019 | 16.363^*^ | .845 | .000 | 14.71 | 18.02 |
|  |  | 2020 | 16.363^*^ | .845 | .000 | 14.71 | 18.02 |
|  |  | 2022 | .274 | .845 | .746 | -1.38 | 1.93 |
|  | 2022 | 2019 | 16.090^*^ | .845 | .000 | 14.43 | 17.75 |
|  |  | 2020 | 16.090^*^ | .845 | .000 | 14.43 | 17.75 |
|  |  | 2021 | -.274 | .845 | .746 | -1.93 | 1.38 |
| pull-ups | 2019 | 2020 | .000 | .141 | 1.000 | -.28 | .28 |
|  |  | 2021 | -.062 | .141 | .660 | -.34 | .21 |
|  |  | 2022 | -.079 | .141 | .577 | -.36 | .20 |
|  | 2020 | 2019 | .000 | .141 | 1.000 | -.28 | .28 |
|  |  | 2021 | -.062 | .141 | .660 | -.34 | .21 |
|  |  | 2022 | -.079 | .141 | .577 | -.36 | .20 |
|  | 2021 | 2019 | .062 | .141 | .660 | -.21 | .34 |
|  |  | 2020 | .062 | .141 | .660 | -.21 | .34 |
|  |  | 2022 | -.017 | .141 | .907 | -.29 | .26 |
|  | 2022 | 2019 | .079 | .141 | .577 | -.20 | .36 |
|  |  | 2020 | .079 | .141 | .577 | -.20 | .36 |
|  |  | 2021 | .017 | .141 | .907 | -.26 | .29 |
| *. The significance level for the difference in means is 0.05.  LSD: Least Significant Difference | | | | | | | |

| **One-way ANOVA (female)** | | | | | | |
| --- | --- | --- | --- | --- | --- | --- |
|  | | square sum | df | mean square | F | significance |
| weight | Intergroup | 2196.729 | 3 | 732.243 | 11.446 | .000 |
|  | Within Groups | 820421.274 | 12824 | 63.975 |  |  |
|  | Total | 822618.003 | 12827 |  |  |  |
| BMI | Intergroup | 199.468 | 3 | 66.489 | 8.269 | .000 |
|  | Within Groups | 103118.110 | 12824 | 8.041 |  |  |
|  | Total | 103317.578 | 12827 |  |  |  |
| Vital capacity | Intergroup | 327522471.578 | 3 | 109174157.193 | 941.837 | .000 |
|  | Within Groups | 1486509521.959 | 12824 | 115916.214 |  |  |
|  | Total | 1814031993.537 | 12827 |  |  |  |
| 50-m run | Intergroup | 558.931 | 3 | 186.310 | 1187.223 | .000 |
|  | Within Groups | 2012.464 | 12824 | .157 |  |  |
|  | Total | 2571.395 | 12827 |  |  |  |
| long-jump | Intergroup | 513330.072 | 3 | 171110.024 | 988.040 | .000 |
|  | Within Groups | 2220877.600 | 12824 | 173.181 |  |  |
|  | Total | 2734207.672 | 12827 |  |  |  |
| sit-reach | Intergroup | 16090.773 | 3 | 5363.591 | 144.360 | .000 |
|  | Within Groups | 476467.451 | 12824 | 37.154 |  |  |
|  | Total | 492558.224 | 12827 |  |  |  |
| 800-m run | Intergroup | 10302.822 | 3 | 3434.274 | 11.161 | .000 |
|  | Within Groups | 3945971.338 | 12824 | 307.702 |  |  |
|  | Total | 3956274.160 | 12827 |  |  |  |
| sit-ups | Intergroup | 89947.581 | 3 | 29982.527 | 912.353 | .000 |
|  | Within Groups | 421433.381 | 12824 | 32.863 |  |  |
|  | Total | 511380.963 | 12827 |  |  |  |

Notes: ANOVA:Analysis of Variance

| **Multiple comparisons（LSD）female** | | | | | | | | |
| --- | --- | --- | --- | --- | --- | --- | --- | --- |
| Dependent Variable | (I) year | | (J) year | Mean Deviation (I-J) | standard error | significance | 95% CI | |
|  |  |  |  |  |  |  | lower-bound | upper-bound |
| height | 2019 | | 2020 | .000 | .138 | 1.000 | -.27 | .27 |
|  |  |  | 2021 | -.415^*^ | .138 | .003 | -.69 | -.15 |
|  |  |  | 2022 | -.494^*^ | .138 | .000 | -.76 | -.22 |
|  | 2020 | | 2019 | .000 | .138 | 1.000 | -.27 | .27 |
|  |  |  | 2021 | -.415^*^ | .138 | .003 | -.69 | -.15 |
|  |  |  | 2022 | -.494^*^ | .138 | .000 | -.76 | -.22 |
|  | 2021 | | 2019 | .415^*^ | .138 | .003 | .15 | .69 |
|  |  |  | 2020 | .415^*^ | .138 | .003 | .15 | .69 |
|  |  |  | 2022 | -.079 | .138 | .569 | -.35 | .19 |
|  | 2022 | | 2019 | .494^*^ | .138 | .000 | .22 | .76 |
|  |  |  | 2020 | .494^*^ | .138 | .000 | .22 | .76 |
|  | |  | 2021 | .079 | .138 | .569 | -.19 | .35 |
| weight | | 2019 | 2020 | .044 | .200 | .824 | -.35 | .44 |
|  |  |  | 2021 | .008 | .200 | .968 | -.38 | .40 |
|  |  |  | 2022 | -.937^*^ | .200 | .000 | -1.33 | -.55 |
|  |  | 2020 | 2019 | -.044 | .200 | .824 | -.44 | .35 |
|  |  |  | 2021 | -.036 | .200 | .856 | -.43 | .36 |
|  |  |  | 2022 | -.982^*^ | .200 | .000 | -1.37 | -.59 |
|  |  | 2021 | 2019 | -.008 | .200 | .968 | -.40 | .38 |
|  |  | | 2020 | .036 | .200 | .856 | -.36 | .43 |
|  |  |  | 2022 | -.945^*^ | .200 | .000 | -1.34 | -.55 |
|  | 2022 | | 2019 | .937^*^ | .200 | .000 | .55 | 1.33 |
|  |  |  | 2020 | .982^*^ | .200 | .000 | .59 | 1.37 |
|  |  |  | 2021 | .945^*^ | .200 | .000 | .55 | 1.34 |
| BMI | 2019 | | 2020 | .029 | .071 | .682 | -.11 | .17 |
|  |  |  | 2021 | .107 | .071 | .132 | -.03 | .25 |
|  |  |  | 2022 | -.228^*^ | .071 | .001 | -.37 | -.09 |
|  | 2020 | | 2019 | -.029 | .071 | .682 | -.17 | .11 |
|  |  |  | 2021 | .078 | .071 | .273 | -.06 | .22 |
|  |  |  | 2022 | -.257^*^ | .071 | .000 | -.40 | -.12 |
|  | 2021 | | 2019 | -.107 | .071 | .132 | -.25 | .03 |
|  |  |  | 2020 | -.078 | .071 | .273 | -.22 | .06 |
|  |  |  | 2022 | -.335^*^ | .071 | .000 | -.47 | -.20 |
|  | 2022 | | 2019 | .228^*^ | .071 | .001 | .09 | .37 |
|  |  |  | 2020 | .257^*^ | .071 | .000 | .12 | .40 |
|  |  |  | 2021 | .335^*^ | .071 | .000 | .20 | .47 |
| Vital capacity | 2019 | | 2020 | -1.818 | 8.502 | .831 | -18.48 | 14.85 |
|  |  |  | 2021 | -375.417^*^ | 8.502 | .000 | -392.08 | -358.75 |
|  |  |  | 2022 | -18.529^*^ | 8.502 | .029 | -35.19 | -1.86 |
|  | 2020 | | 2019 | 1.818 | 8.502 | .831 | -14.85 | 18.48 |
|  |  |  | 2021 | -373.599^*^ | 8.502 | .000 | -390.26 | -356.93 |
|  |  |  | 2022 | -16.711^*^ | 8.502 | .049 | -33.38 | -.04 |
|  | 2021 | | 2019 | 375.417^*^ | 8.502 | .000 | 358.75 | 392.08 |
|  |  |  | 2020 | 373.599^*^ | 8.502 | .000 | 356.93 | 390.26 |
|  |  |  | 2022 | 356.888^*^ | 8.502 | .000 | 340.22 | 373.55 |
|  | 2022 | | 2019 | 18.529^*^ | 8.502 | .029 | 1.86 | 35.19 |
|  |  |  | 2020 | 16.711^*^ | 8.502 | .049 | .04 | 33.38 |
|  |  |  | 2021 | -356.888^*^ | 8.502 | .000 | -373.55 | -340.22 |
| 50-m run | 2019 | | 2020 | .000 | .010 | 1.000 | -.02 | .02 |
|  |  |  | 2021 | .010 | .010 | .315 | -.01 | .03 |
|  |  |  | 2022 | .485^*^ | .010 | .000 | .47 | .50 |
|  | 2020 | | 2019 | .000 | .010 | 1.000 | -.02 | .02 |
|  |  |  | 2021 | .010 | .010 | .315 | -.01 | .03 |
|  |  |  | 2022 | .485^*^ | .010 | .000 | .47 | .50 |
|  | 2021 | | 2019 | -.010 | .010 | .315 | -.03 | .01 |
|  |  |  | 2020 | -.010 | .010 | .315 | -.03 | .01 |
|  |  |  | 2022 | .475^*^ | .010 | .000 | .46 | .49 |
|  | 2022 | | 2019 | -.485^*^ | .010 | .000 | -.50 | -.47 |
|  |  |  | 2020 | -.485^*^ | .010 | .000 | -.50 | -.47 |
|  |  |  | 2021 | -.475^*^ | .010 | .000 | -.49 | -.46 |
| long-jump | 2019 | | 2020 | -1.253^*^ | .329 | .000 | -1.90 | -.61 |
|  |  |  | 2021 | -14.747^*^ | .329 | .000 | -15.39 | -14.10 |
|  |  |  | 2022 | .594 | .329 | .071 | -.05 | 1.24 |
|  | 2020 | | 2019 | 1.253^*^ | .329 | .000 | .61 | 1.90 |
|  |  |  | 2021 | -13.495^*^ | .329 | .000 | -14.14 | -12.85 |
|  |  |  | 2022 | 1.847^*^ | .329 | .000 | 1.20 | 2.49 |
|  | 2021 | | 2019 | 14.747^*^ | .329 | .000 | 14.10 | 15.39 |
|  |  |  | 2020 | 13.495^*^ | .329 | .000 | 12.85 | 14.14 |
|  |  |  | 2022 | 15.341^*^ | .329 | .000 | 14.70 | 15.99 |
|  | 2022 | | 2019 | -.594 | .329 | .071 | -1.24 | .05 |
|  |  |  | 2020 | -1.847^*^ | .329 | .000 | -2.49 | -1.20 |
|  |  |  | 2021 | -15.341^*^ | .329 | .000 | -15.99 | -14.70 |
| sit-reach | 2019 | | 2020 | .000 | .152 | 1.000 | -.30 | .30 |
|  |  |  | 2021 | 1.126^*^ | .152 | .000 | .83 | 1.42 |
|  |  |  | 2022 | 2.734^*^ | .152 | .000 | 2.44 | 3.03 |
|  | 2020 | | 2019 | .000 | .152 | 1.000 | -.30 | .30 |
|  |  |  | 2021 | 1.126^*^ | .152 | .000 | .83 | 1.42 |
|  |  |  | 2022 | 2.734^*^ | .152 | .000 | 2.44 | 3.03 |
|  | 2021 | | 2019 | -1.126^*^ | .152 | .000 | -1.42 | -.83 |
|  |  |  | 2020 | -1.126^*^ | .152 | .000 | -1.42 | -.83 |
|  |  |  | 2022 | 1.608^*^ | .152 | .000 | 1.31 | 1.91 |
|  | 2022 | | 2019 | -2.734^*^ | .152 | .000 | -3.03 | -2.44 |
|  |  |  | 2020 | -2.734^*^ | .152 | .000 | -3.03 | -2.44 |
|  |  |  | 2021 | -1.608^*^ | .152 | .000 | -1.91 | -1.31 |
| 800-m run | 2019 | | 2020 | .000 | .438 | 1.000 | -.86 | .86 |
|  |  |  | 2021 | -1.904^*^ | .438 | .000 | -2.76 | -1.05 |
|  |  |  | 2022 | -1.665^*^ | .438 | .000 | -2.52 | -.81 |
|  | 2020 | | 2019 | .000 | .438 | 1.000 | -.86 | .86 |
|  |  |  | 2021 | -1.904^*^ | .438 | .000 | -2.76 | -1.05 |
|  |  |  | 2022 | -1.665^*^ | .438 | .000 | -2.52 | -.81 |
|  | 2021 | | 2019 | 1.904^*^ | .438 | .000 | 1.05 | 2.76 |
|  |  |  | 2020 | 1.904^*^ | .438 | .000 | 1.05 | 2.76 |
|  |  |  | 2022 | .239 | .438 | .585 | -.62 | 1.10 |
|  | 2022 | | 2019 | 1.665^*^ | .438 | .000 | .81 | 2.52 |
|  |  |  | 2020 | 1.665^*^ | .438 | .000 | .81 | 2.52 |
|  |  |  | 2021 | -.239 | .438 | .585 | -1.10 | .62 |
| sit-ups | 2019 | | 2020 | -.152 | .143 | .290 | -.43 | .13 |
|  |  |  | 2021 | -4.478^*^ | .143 | .000 | -4.76 | -4.20 |
|  |  |  | 2022 | -6.033^*^ | .143 | .000 | -6.31 | -5.75 |
|  | 2020 | | 2019 | .152 | .143 | .290 | -.13 | .43 |
|  |  |  | 2021 | -4.326^*^ | .143 | .000 | -4.61 | -4.05 |
|  |  |  | 2022 | -5.881^*^ | .143 | .000 | -6.16 | -5.60 |
|  | 2021 | | 2019 | 4.478^*^ | .143 | .000 | 4.20 | 4.76 |
|  |  |  | 2020 | 4.326^*^ | .143 | .000 | 4.05 | 4.61 |
|  |  |  | 2022 | -1.555^*^ | .143 | .000 | -1.84 | -1.27 |
|  | 2022 | | 2019 | 6.033^*^ | .143 | .000 | 5.75 | 6.31 |
|  |  |  | 2020 | 5.881^*^ | .143 | .000 | 5.60 | 6.16 |
|  |  |  | 2021 | 1.555^*^ | .143 | .000 | 1.27 | 1.84 |
| *. The significant level of mean deviation is 0.05. LSD: Least Significant Difference | | | | | | | | |
