# Supplementary material for: A longitudinal study of four-year changes in physical fitness among university students before and after COVID-19: 2019–2022
Source: PLoS One. 2025 Oct 10;20(10):e0334088. doi: 10.1371/journal.pone.0334088 (PMC12513638; doi:10.1371/journal.pone.0334088)
Supplement: Attachment — Submitted filename: PONE-D-25-29142 - rev.pdf [file pone.0334088.s002.pdf]

PLOS ONE

A longitudinal study of four-year changes in physical fitness among university students before and after COVID-19: 2019-2022

--Manuscript Draft--

Manuscript Number:	PONE-D-25-29142
Article Type:	Research Article
Full Title:	A longitudinal study of four-year changes in physical fitness among university students before and after COVID-19: 2019-2022
Short Title:	Four-year fitness changes in university students pre/post-COVID-19
Corresponding Author:	Liang Jiang<br>Hengyang Normal University<br>CHINA
Keywords:	COVID-19; physical fitness; body shape; university students; Longitudinal study
Abstract:	Background<br><br>Physical fitness has been widely recognized as a strong marker of health in children and adolescents and has been negatively impacted by the COVID-19 pandemic. Few studies have conducted longitudinal analyses of changes in physical fitness due to the impact of the pandemic over a four-year period in university for freshman students enrolled in 2019 (i.e., prior to the COVID-19 pandemic). Such longitudinal studies are critical to understanding the long-term effects of the pandemic on university students' body shape, physical fitness, and prevalence of overweight and obesity, particularly with respect to temporal and gender differences.<br><br>Methods<br><br>We recruited 4,413 university students (males: n=1,206, 27%; females: n=3,207, 73%) from a Chinese university for this four-year cohort study. From 2019 through 2022 (before, during and after the pandemic), all subjects participated in the same physical fitness tests. A total of 10 physical fitness measures were tested: height, weight, vital capacity, long-jump, sit-reach, 50-m run, 1,000-m run and pull-ups (boys) and 800-m run and sit-ups (girls). Data were analyzed using one-way ANOVA, LSD test, Chi-squared test and scatterplot3dimensional correlation.<br><br>Results<br><br>Results revealed significant increases in weight and obesity rates, with male obesity rates rising from 6.97% to 11.6% and female rates from 1.9% to 3.45%. Overweight prevalence also increased slightly in both genders. vital capacity peaked in 2021 but dropped sharply in 2022 for both males (4,114.16 mL to 3,934.31 mL) and females (3,314.69 mL to 2,957.8 mL). Long-jump and sit-reach scores decreased over time, and cardiorespiratory endurance (1,000-m run for males, 800-m run for females) worsened in 2021-2022 compared to 2019-2020. However, female students showed improvements in 50-m run times and sit-ups performance, while male students exhibited no significant changes in 50-m run or pull-ups.<br><br>Conclusions<br><br>Significant physical fitness changes occurred during the four years of university due to the impact of the pandemic. School policies and physical education teachers should focus more on controlling the body mass index in all adolescents and reducing the increase in overweight and obesity rates, especially in male university students. Focus should be placed on the ability of university students to develop agility, explosiveness, and muscular strength at each stage and on improving cardiorespiratory endurance in the third and fourth years of university students.
Order of Authors:	Liangru Guo
	Liang Jiang

Powered by Editorial Manager® and ProduXion Manager® from Aries Systems Corporation

	Huizhi Huang
**Additional Information:**	
**Question**	**Response**
**Financial Disclosure**<br><br>Enter a financial disclosure statement that describes the sources of funding for the work included in this submission. Review the submission guidelines for detailed requirements. View published research articles from *PLOS ONE* for specific examples.<br><br>This statement is required for submission and **will appear in the published article** if the submission is accepted. Please make sure it is accurate.<br><br>**Funded studies**<br>Enter a statement with the following details:<br>• Initials of the authors who received each award<br>• Grant numbers awarded to each author<br>• The full name of each funder<br>• URL of each funder website<br>• Did the sponsors or funders play any role in the study design, data collection and analysis, decision to publish, or preparation of the manuscript?<br><br>Did you receive funding for this work?	Yes
Please add funding details.<br>as follow-up to "**Financial Disclosure**<br><br>Enter a financial disclosure statement that describes the sources of funding for the work included in this submission. Review the submission guidelines for detailed requirements. View published research articles from *PLOS ONE* for specific examples.<br><br>This statement is required for submission and **will appear in the published article** if the submission is accepted. Please make sure it is accurate.	This research was funded by the Research Project on Teaching Reform of General Colleges and Universities in Hunan Province in 2023 (Project No. HNJG-20230872), the Fourteenth Five-Year Plan of Educational Science in Hunan Province in 2024 (Project No. XJK24CTW013), and the Deepening Educational Evaluation Reform in the New Era in Hunan Province in 2022 Pilot Project (Project No. TGYX202207).

**Funded studies**<br>Enter a statement with the following details:<br>• Initials of the authors who received each award<br>• Grant numbers awarded to each author<br>• The full name of each funder<br>• URL of each funder website<br>• Did the sponsors or funders play any role in the study design, data collection and analysis, decision to publish, or preparation of the manuscript?<br><br>Did you receive funding for this work?"	
Please select the country of your main research funder (please select carefully as in some cases this is used in fee calculation).<br>as follow-up to "**Financial Disclosure**<br><br>Enter a financial disclosure statement that describes the sources of funding for the work included in this submission. Review the submission guidelines for detailed requirements. View published research articles from *PLOS ONE* for specific examples.<br><br>This statement is required for submission and **will appear in the published article** if the submission is accepted. Please make sure it is accurate.<br><br>**Funded studies**<br>Enter a statement with the following details:<br>• Initials of the authors who received each award<br>• Grant numbers awarded to each author<br>• The full name of each funder<br>• URL of each funder website<br>• Did the sponsors or funders play any role in the study design, data collection and analysis, decision to publish, or preparation of the manuscript?<br><br>Did you receive funding for this work?"	CHINA - CN
**Competing Interests**	The authors have declared that no competing interests exist.

Use the instructions below to enter a competing interest statement for this submission. On behalf of all authors, disclose any competing interests that could be perceived to bias this work—acknowledging all financial support and any other relevant financial or non-financial competing interests.<br><br>This statement is required for submission and **will appear in the published article** if the submission is accepted. Please make sure it is accurate and that any funding sources listed in your Funding Information later in the submission form are also declared in your Financial Disclosure statement.<br><br>View published research articles from *PLOS ONE* for specific examples.<br><br>**NO authors have competing interests**<br><br>Enter: *The authors have declared that no competing interests exist.*<br><br>**Authors with competing interests**<br><br>Enter competing interest details beginning with this statement:<br><br>*I have read the journal's policy and the authors of this manuscript have the following competing interests: [insert competing interests here]*<br><br>* typeset	
**Ethics Statement**<br><br>Enter an ethics statement for this submission. This statement is required if the study involved:<br><br>• Human participants<br>• Human specimens or tissue<br>• Vertebrate animals or cephalopods<br>• Vertebrate embryos or tissues	The study was conducted in accordance with the Declaration of Helsinki, and approved by the Institutional Review Board of the School of Sports Science, Hengyang Normal University OF INSTITUTE(September 18, 2019).

Powered by Editorial Manager® and ProduXion Manager® from Aries Systems Corporation

• Field research<br><br>Write "N/A" if the submission does not require an ethics statement.<br><br>General guidance is provided below. Consult the submission guidelines for detailed instructions. **Make sure that all information entered here is included in the Methods section of the manuscript.**	

Powered by Editorial Manager® and ProduXion Manager® from Aries Systems Corporation

**Format for specific study types**<br><br>**Human Subject Research (involving human participants and/or tissue)**<br>• Give the name of the institutional review board or ethics committee that approved the study<br>• Include the approval number and/or a statement indicating approval of this research<br>• Indicate the form of consent obtained (written/oral) or the reason that consent was not obtained (e.g. the data were analyzed anonymously)<br><br>**Animal Research (involving vertebrate animals, embryos or tissues)**<br>• Provide the name of the Institutional Animal Care and Use Committee (IACUC) or other relevant ethics board that reviewed the study protocol, and indicate whether they approved this research or granted a formal waiver of ethical approval<br>• Include an approval number if one was obtained<br>• If the study involved *non-human primates*, add *additional details* about animal welfare and steps taken to ameliorate suffering<br>• If anesthesia, euthanasia, or any kind of animal sacrifice is part of the study, include briefly which substances and/or methods were applied<br><br>**Field Research**<br><br>Include the following details if this study involves the collection of plant, animal, or other materials from a natural setting:<br>• Field permit number<br>• Name of the institution or relevant body that granted permission	
**Data Availability**<br><br>Authors are required to make all data underlying the findings described fully available, without restriction, and from the time of publication. PLOS allows rare exceptions to address legal and ethical concerns. See the PLOS Data Policy and FAQ for detailed information.	Yes - all data are fully available without restriction

A Data Availability Statement describing where the data can be found is required at submission. Your answers to this question constitute the Data Availability Statement and **will be published in the article**, if accepted.<br><br>**Important:** Stating 'data available on request from the author' is not sufficient. If your data are only available upon request, select 'No' for the first question and explain your exceptional situation in the text box.<br><br>Do the authors confirm that all data underlying the findings described in their manuscript are fully available without restriction?	
**Describe where the data may be found in full sentences. If you are copying our sample text, replace any instances of XXX with the appropriate details.**<br><br>• If the data are **held or will be held in a public repository**, include URLs, accession numbers or DOIs. If this information will only be available after acceptance, indicate this by ticking the box below. For example: *All XXX files are available from the XXX database (accession number(s) XXX, XXX.*).<br>• If the data are all contained **within the manuscript and/or Supporting Information files**, enter the following: *All relevant data are within the manuscript and its Supporting Information files.*<br>• If neither of these applies but you are able to provide **details of access elsewhere**, with or without limitations, please do so. For example:<br><br>*Data cannot be shared publicly because of [XXX]. Data are available from the XXX Institutional Data Access / Ethics Committee (contact via XXX) for researchers who meet the criteria for access to confidential data.*<br><br>*The data underlying the results presented in the study are available from (include the name of the third party*	All relevant data are within the manuscript and its Supporting Information files.

and contact information or URL).<br>• This text is appropriate if the data are owned by a third party and authors do not have permission to share the data.<br><br>* typeset	
Additional data availability information:	

Manuscript Click here to access/download;Manuscript;Manuscript.docx

A longitudinal study of four-year changes in physical fitness among university students before and after COVID-19: 2019-2022

Liangru Guo[1], Liang Jiang [1*], and Huizhi Huang[1]

1 School of Sports Science, Hengyang Normal University, Hengyang , Hunan Province 421010, China.

[*] **Correspondence:**

Corresponding Liang Jiang

ll1553609@163.com

Keywords: COVID-19; physical fitness; body shape; university students; longitudinal study

Abstract

Background: Physical fitness has been widely recognized as a strong marker of health in children and adolescents and has been negatively impacted by the COVID-19 pandemic. Few studies have conducted longitudinal analyses of changes in physical fitness due to the impact of the pandemic over a four-year period in university for freshman students enrolled in 2019 (i.e., prior to the COVID-19 pandemic). Such longitudinal studies are critical to understanding the long-term effects of the pandemic on university students' body shape, physical fitness, and prevalence of overweight and obesity, particularly with respect to temporal and gender differences. **Methods:** We recruited 4,413 university students (males: n=1,206, 27%; females: n=3,207, 73%) from a Chinese university for this four-year cohort study. From 2019 through 2022 (before, during and after the pandemic), all subjects participated in the same physical fitness tests. A total of 10 physical fitness measures were tested: height, weight, vital capacity, long-jump, sit-reach, 50-m run, 1,000-m run and pull-ups (boys) and 800-m run and sit-ups (girls). Data were analyzed using one-way ANOVA, LSD test, Chi-squared test and scatterplot3dimensional correlation. **Results:** Results revealed significant increases in weight and obesity rates, with male obesity rates rising from 6.97% to 11.6% and female rates from 1.9% to 3.45%. Overweight prevalence also increased slightly in both genders. vital capacity peaked in 2021 but dropped sharply in 2022 for both males (4,114.16 mL to 3,934.31 mL) and females (3,314.69 mL to 2,957.8 mL). Long-jump and sit-reach scores decreased

over time, and cardiorespiratory endurance (1,000-m run for males, 800-m run for females) worsened in 2021-2022 compared to 2019-2020. However, female students showed improvements in 50-m run times and sit-ups performance, while male students exhibited no significant changes in 50-m run or pull-ups.**Conclusions:** Significant physical fitness changes occurred during the four years of university due to the impact of the pandemic. School policies and physical education teachers should focus more on controlling the body mass index in all adolescents and reducing the increase in overweight and obesity rates, especially in male university students. Focus should be placed on the ability of university students to develop agility, explosiveness, and muscular strength at each stage and on improving cardiorespiratory endurance in the third and fourth years of university students.

1 Introduction

Coronavirus Disease 2019 (COVID-19) was first identified in 2019 and subsequently spread rapidly to most countries around the world in just a few months, becoming a global public health crisis [1]. The pandemic has not only put enormous pressure on global healthcare systems, but has also had a profound impact on human physical and mental health. An international online survey study covering multiple countries showed that home quarantine measures during the COVID-19 pandemic had a significant negative impact on all physical activity (PA) intensity levels, including vigorous, moderate, walking, and overall activity. In addition, it was found that

people's daily sitting time increased significantly from an average of 5 to 8 hours during the pandemic, and that this increase in sedentary behavior severely impacted the public's level of physical fitness [2]. The university years constitute a critical period of transition from adolescence to adulthood and an important stage for individuals to form healthy lifestyles and establish healthy behavior patterns [3]. However, the outbreak of the COVID-19 pandemic and the resultant prevention and control measures have significantly disrupted this process. In response to the pandemic, the Chinese government adopted a series of strict preventive and control measures, including closing schools and quickly introducing online teaching as an alternative. From December 2019, mass online teaching was implemented nationwide until September 2020, when offline teaching was gradually restored. While social distancing and lockdown policies proved to be effective means of curbing the spread of the virus, these measures also drastically altered the public's daily lifestyle, with far-reaching effects on the health behaviors of university students in particular. Prolonged periods of online learning and school closures have led to an unintended consequence in the form of a dramatic decline in physical activity levels among the university students. Numerous studies have shown that physical activity is negatively correlated with the incidence of several chronic diseases, including high cholesterol [4], obesity [5], cancer [6] ,hypertension and diabetes [7]. Reduced physical activity during the pandemic may have exacerbated these health risks, especially among the key population of university students. In addition, studies have noted that increased

psychological stress and social distancing during the pandemic may have further exacerbated the deterioration of health behaviors [8].

Declining physical fitness has become a global social problem that has been further exacerbated, especially during the COVID-19 pandemic [9]. Several studies worldwide have focused on how the restrictions resulting from the COVID-19 pandemic exerted impacts on the physical fitness of children and adolescents, with the vast majority of findings suggesting significant negative impacts on physical fitness due to decreased physical activity and increased sedentary behavior [10-14]. However, recent studies have also revealed the complexity of this issue. One of these studies showed that not all health indicators were negatively affected by the lockdown, with body mass index (BMI), muscle strength, and flexibility qualities being the main ones affected [15]; another study pointed out that Chinese university students experienced an increase in BMI both during and after the lockdown [16]. These two studies are important references: the first study conducted a one-year follow-up of the effects of the COVID-19 lockdown on BMI and physical fitness of Chinese university students; the second study analyzed the changes in BMI mainly over a three-year follow-up period. Although these studies provide valuable insights, there are still research gaps. In particular, few studies have conducted longitudinal analyses of changes in body mass due to the impact of the pandemic over a four-year period in university for freshman students enrolled in 2019 (i.e., prior to the COVID-19 pandemic). Such longitudinal studies are critical to understanding the long-term effects of the pandemic

on university students' body shape, physical fitness, and prevalence of overweight and obesity, particularly with respect to temporal and gender differences. Therefore, this study analyzed changes in body shape, physical fitness, and prevalence of overweight and obesity among university students before and after the COVID-19 pandemic through longitudinal in-depth analyses, with particular attention to temporal and gender differences. It aims to pay more attention to the long-term impact of pandemic prevention and control measures on public health behaviors, especially on the university students, when formulating future public health policies.

2 Materials and methods

2.1 Study population

The recruitment period for this study spanned from September 5, 2019, to September 28, 2019. A total of 4,413 subjects, including 1,206 male and 3,207 female students, were selected from freshmen enrolled during this period at a teacher training university in Hunan Province and were followed up for four years throughout their university study. According to the “China Student Physical Fitness and Health Research Workbook” [17], the physical fitness and health data of these university students were investigated in 2019 (freshman stage, before the pandemic), 2020 (sophomore stage, during the pandemic), 2021 (junior stage, after the pandemic), and 2022 (senior stage, after the pandemic). Oral informed consent was obtained from all subjects prior to their inclusion in this study.

2.2 Physical fitness test

Physical fitness tests for both boys and girls included height, weight, vital capacity, long-jump, sit-reach, and 50-m run. There were also pull-ups and a 1,000-m run for boys and an 800-m run and sit-ups for girls. Physical fitness tests were administered by physical education professors and conducted annually from October through November, and before taking such test, participants were required to warm up.

2.2.1 Vital capacity

The vital capacity was measured using an air spirometer with a dry and sterilized plastic mouthpiece. Participants took a deep breath and slowly exhaled into the mouthpiece until they could no longer exhale. The air spirometer automatically calculated the maximum air volume (vital capacity per milliliter) and displayed the result when the blowing was complete. Participants took the test twice at an interval of 15 s and the better of the two scores was recorded.

2.2.2 Long-jump

Participants were asked to stand behind the starting line with their feet apart and jump forward in a natural position. Participants were also instructed that both feet should jump at the same time from a standing position and that no additional movements should be made. Horizontal distance was measured from the trailing edge of the

starting point to the trailing edge of the nearest landing point. Each student was allowed for three attempts. The longest jump was recorded in centimeters.

2.2.3 Sit-reach

Participants were tested for sitting and stretching using a seated stretch box device, in a seated position and keeping their legs straight. The participant's upper body was leaned forward, with his/her arms straightened forward and his/her feet separated by 10 to 15 centimeters while pushing horizontally forward against the test board. The participant's fingertips extended and gradually pushed the test bar forward until he/she was unable to push further. Each student was given two attempts. The farthest distance was recorded in centimeters.

2.2.4 Pull-ups

The test is aimed to check a participant's upper body strength. The participant held the bar in a shoulder-width hand to form a straight-arm suspension position, and then pulled the entire weight upward simultaneously using only the arms while keeping the body and arms straight. In addition, the participant's body could not make additional movements, such as swinging or oscillating. In order to complete a pull-up, the participants were required to pull themselves up until their jaws were beyond the upper edge of the barbell. The number of pull-ups was recorded.

2.2.5 50-m run

The test is aimed to measure a participant's explosive power and highest speed. Participants were directed to the starting line of a 50-meter straightaway and were placed in a standing position. In addition, participants were instructed to start responding to the coach's word “ready-and-go”. The timekeeper began recording the time according to the coach's instructions. The time was recorded as the moment the participant crossed the finish line with his/her chest. The recoded time is in seconds and is accurate to one decimal place. The second digit after the decimal point was rounded to 1 according to the non-zero principle. Each participant was given two attempts and the better performance was recorded.

2.2.6 1,000-m run

The test measures a participant's aerobic endurance. Participants were asked to stand behind the starting line of the 1,000-meter run. The test began with a ready-and-go instruction from the coach. Upon the coach’ s such instruction, the timekeeper began recording. Then, when the participant crossed the finish line, the timekeeper finished recording. Time was recoded in minutes and seconds to one decimal place. The second digit after the decimal point was rounded up to 1 according to the non-zero principle. Each participant had only one chance to take the test.

2.2.7 Body mass index (BMI)

BMI was used to measure the degree of overweight and obesity of the participants, expressed as weight (kg)/height (m)2. The WHO-defined BMI criteria were used,

with a BMI ≥ 28 (kg/m2) defined as obesity and 24 ≤ BMI < 27.9 defined as overweight. Participants' height and weight were measured by using electronic weight and height scales. Participants were instructed to remove their shoes and stand up straight to step on the electronic scale. The electronic scale then automatically measured the individual's weight and height to the nearest 0.1 cm.

2.3 Statistical analysis

Data were initially entered using Excel 2016 and statistically analyzed using the software SPSS 27.0; quantitative data were expressed using mean ± standard deviation. Comparisons between adjacent years stratified by gender were performed using one-way analysis of variance (ANOVA), which was used to check for differences; χ^2 test in SPSS 27.0 was used to analyze differences in the detection rates of overweight and obesity in the same year for different gender groups; and χ^2 linear trend test was used to analyze the trends in the detection rates of overweight and obesity in the whole population, both genders, for the years 2019-2022. The annual growth rate (%/year) of the detection rate of each gender-age group in different periods was calculated (or folded): Annual growth rate=(detection rate in a certain year - detection rate in the previous year)/(a certain year - the previous year)×100%. A two-sided test was used, and a difference of $P<0.05$ was considered statistically significant. The scatterplot3d package in the software R 3.0.0 was used to obtain 3D scatterplots.

3 Results

3.1 Demographic characteristics of participants

Table 1 summarizes the demographic characteristics of the participants: female students outnumbered male students (72.7% to 27.3%); their age was calculated based on the enrollment in September 2019, with the 7 ≤ YEAR ≤ 19 year old age group accounting for 91.9% of the total participants, followed by the < 17 year old age group accounting for 4.7% and the > 19 year old age group accounting for 3.4%; 89.2% of the university students were Han Chinese and 10.8% were ethnic minorities.

Table 1. Demographic characteristics

Variable	Category	Frequency	Percent valid	Effective percentage	Cumulative percentage
Gender	Male	1,206	27.3	27.3	27.3
	Female	3,207	72.7	72.7	100.0
Age	<17	208	4.7	4.7	4.7
	17≤year≤19	4,055	91.9	91.9	96.6
	＞19	150	3.4	3.4	100.0
Ethnicity	Han Chinese	3,935	89.2	89.2	89.2
	Ethnic minority	478	10.8	10.8	100.0

3.2. Results of ANOVA for university students' physical fitness

3.2.1 Changes in body shape

In terms of height changes, the male group did not show a significant difference in the mean height between the four years (F=1.745, p>0.05). In contrast, the female group showed a significant difference in the mean height (F=7.375, p<0.01). The LSD test, a post-hoc test, showed that the difference in the height of the females was not significant between 2019 and 2020, nor between 2021 and 2022, but those in the latter two years (2021-2022) were significantly higher than those in the first two years (2019-2020). (Table 2)

Regarding weight changes, there was a significant difference in the mean weight of the male group (F=19.698, p<0.01). LSD analysis showed that there was no significant difference in weight between 2019 and 2020, but there was a trend of increasing year by year from 2021 to 2022. The same significant difference was found in the females (F=11.446, p<0.01), whose weight remained stable between 2019, 2020 and 2021, but was significantly higher in 2022 than those in the previous three years. (Table 2)

In terms of BMI, there was a significant difference in the males (F=20.095, p<0.01), whose BMI remained stable between 2019-2021 and was significantly higher in 2022. A similar trend was observed in the females (F=8.269, p<0.01), whose BMI did not change significantly between 2019, 2020 and 2021 and was significantly higher in 2022 than those in the previous three years. (Table 2)

Table 2. Results of ANOVA

Variable	Gender	N	2019	2020	2021	2022	F	LSD
Height	Male	1206	172.5 (6.147)	172.5 (6.147)	172.93 (6.216)	172.89 (6.2)	1.745	
	Female	3207	160.15 (5.483)	160.15 (5.483)	160.56 (5.616)	160.64 (5.505)	7.355**	1&2<3&4
Weight	Male	1206	65.75 (12.331)	65.75 (12.331)	66.91 (12.676)	69.16 (12.946)	19.698**	1&2<3<4
	Female	3207	53.96 (7.533)	53.92 (7.738)	53.96 (7.94)	54.9 (8.731)	11.446**	1&2&3<4
BMI	Male	1206	22.06 (3.746)	21.07 (2.67)	20.94 (2.788)	21.22 (3.007)	20.095**	1&2&3<4
	Female	3207	21.38 (3.169)	21 (2.677)	21.97 (3.57)	21.26 (3.104)	8.269**	1&2&3<4
Vital	Male	1206	3880.37 (475.945)	3778.78 (459.555)	4114.16 (446.118)	3934.31 (283.977)	132.75**	2<1<4<3

capacity	Female	3207	2939.28 (337.189)	2941.09 (336.509)	3314.69 (343.613)	2957.8 (344.471)	941.837**	1&2<4<3
50-m run	Male	1206	7.16 (0.475)	7.16 (0.475)	7.18 (0.46)	7.18 (0.466)	0.69	
	Female	3207	9.39 (0.422)	9.39 (0.422)	9.38 (0.415)	8.91 (0.314)	1,187.223**	4<1&2&3
Long-jump	Male	1206	228.94 (14.678)	218.18 (18.543)	229.19 (18.94)	221.75 (14.173)	128.238**	2<4<3&1
	Female	3207	161.8 (13.328)	163.06 (12.587)	176.55 (13.698)	161.21 (13.001)	988.04**	1&4<2<3
Sit-reach	Male	1206	16.07 (6.437)	16.07 (6.437)	15.49 (7.343)	14.7 (6.845)	11.032**	4<3<1&2
	Female	3207	19.63 (5.998)	19.63 (5.998)	18.5 (6.297)	16.89 (6.084)	144.36**	4<3<1&2
1,000-m-run	Male	1206	236.83 (23.536)	236.83 (23.536)	253.19 (17.567)	252.92 (17.458)	246.005**	1&2<3&4

800-m-run	Female	3207	251.69 (17.853)	251.69 (17.853)	253.6 (17.101)	253.36 (17.346)	11.161**	1&2<3&4
Pull-ups	Male	1206	12.16 (3.528)	12.16 (3.528)	12.23 (3.479)	12.24 (3.348)	0.17	
1-min-sit-ups	Female	3207	35.06 (5.658)	35.21 (5.604)	39.54 (5.784)	41.1 (5.88)	912.353**	1&2<3<4

Note:**P<0.01; 1=2019; 2=2020; 3=2021; 4=2022

3.2.2 Changes in physical fitness

There was a significant difference (F=132.75, p<0.01) in the vital capacity test in the males, whose vital capacity continued to increase between 2019-2021, peaked in 2021, and decreased in 2022. The same significant difference was found in the females (F=941.83, p<0.01), whose vital capacity was lower during 2019-2020 than that in 2022, all significantly lower than the peak level in 2021. (Table 2)

The 50-m run test showed no significant difference in the performance of the male group (F=0.69, p>0.05). There was a significant difference in the female group (F=1,187.2, p<0.01), with the performance in 2022 being significantly better than that in the previous three years but remained stable between 2019-2021. (Table 2)

In the long-jump test, there was a significant difference (F=128.238, $p<0.01$) in the male group, with scores in 2020 lower than that in 2022, both lower than those in 2019 and 2021, with no significant difference between scores in 2019 and 2021. The same significant difference was found in the female group (F=988.04, $p<0.01$), with the scores in 2019 and 2022 being significantly lower than those in 2020, both lower than those in 2021, where there was no significant difference between the scores in 2019 and 2022. (Table 2)

The sit-reach test showed a significant difference (F=11.032, $p<0.01$) in the male group, showing a decreasing trend in performance over time, with no significant difference between 2019 and 2020. There was also a significant difference in the female group (F=144.36, $p<0.01$), showing a similar decreasing trend, with no significant difference between 2019 and 2020. (Table 2)

In the endurance running test, there was a significant difference (F=246.005, $p<0.01$) in the 1,000-m run among the males, showing significantly better performance in 2019 and 2020 than in 2021and 2022, where there was no significant difference between 2019 and 2020 or between 2021 and 2022. There was a significant difference (F=11.161, $p<0.01$) in the 800-m run among the females, showing the same trend, with the performance in 2019 and 2020 being significantly better than that in 2021 and 2022, where there was no significant difference between 2019 and 2020 or between 2021 and 2022. (Table 2)

There was a significant difference in the 1-min-sit-ups test in the female group (F=912.35, p<0.01), with the scores in 2019 and 2020 being significantly lower than those in 2021, both lower than 2022, with no significant difference between 2019 and 2020. (Table 2)

3.3 Detection of overweight and obesity among university students

3.3.1 Detection of obesity

In the longitudinal study on the university students' obesity detection rates, an increasing trend year by year was observed. The obesity detection rate for the males increased from 6.97% in 2019 to 11.6% in 2022, showing a linear increase over time [χ^2 (trend)=18.44, P<0.001)]. The obesity detection rate for the females increased from 1.9% in 2019 to 3.45% in 2022, with a linear increase over time [χ^2 (trend)=19.81, P<0.001)]. The figure for the whole population increased from 3.29% to 5.69%, indicating a linear trend over time [χ^2 (trend)=37.39, P<0.001]. Since the detection rates in 2020 and 2019 were consistent, the BMI values for 2019, 2021, and 2022 were included in the correlation analyses, and the results similarly showed a trend of linear increase in BMI over time for both the males and the females (Figures 1 and 2). In terms of gender stratification, the results of the χ^2 test showed that the differences in obesity detection rates between the male and female groups each year were statistically significant (P<0.01), with the males having a higher obesity detection rate than the females. It is worth noting that the obesity detection rates of the male and female groups did not

show a significant increase in 2020, which was the first year of the COVID-19 pandemic (Table 3).

Table 3. Comparison of obesity detection rates

Groups	2019			2020			2021			2022			χ²(trend)
	Statistical value	Total number of people	Number of detections (detection rate)	Statistical value	Total number of people	Number of detections (detection rate)	Statistical value	Total number of people	Number of detections (detection rate)	Statistical value	Total number of people	Number of detections (detection rate)	
Male		1206	84（6.97%)		1206	84 (5.97%)		1206	102 (8.46%)		1206	140 (11.6%)	18.440**
Fe		32	61		32	60		32	85		32	111	19.

ma le	07	(1.9 %)	07	(1.8 7%)	07	(2.6 5%)	07	(3.4 6%)	810 **
	χ²	70.7**	χ²	72.05**	χ²	72.84**	χ²	108.46**	
the tot al	44 13	145 (3.2 9%)	44 13	144 (3.2 6%)	44 13	187 (4.2 4%)	44 13	251 (5.6 9%)	37. 39* *

Note: **P<0.01

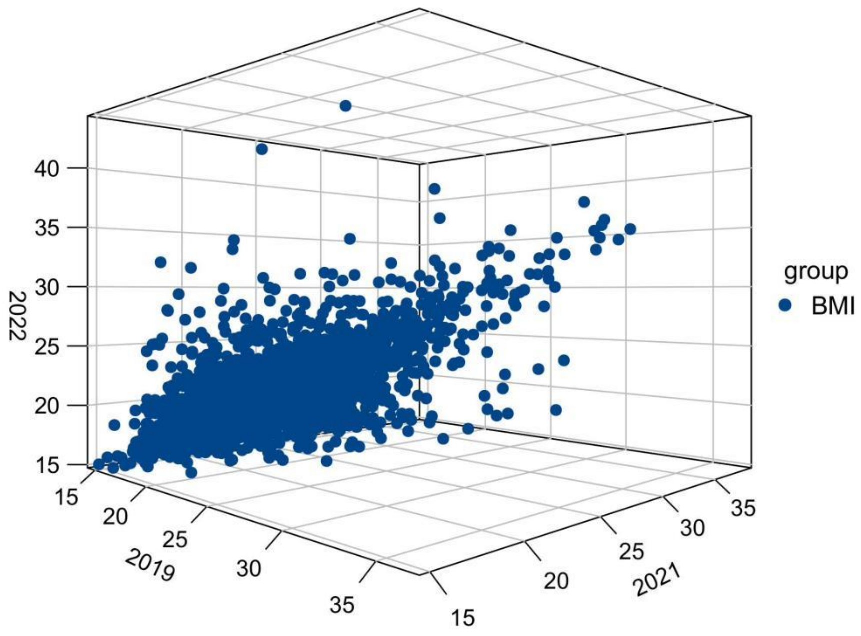

Figure 1. Three-dimensional correlation of BMI in females in 2019, 2021 and 2022

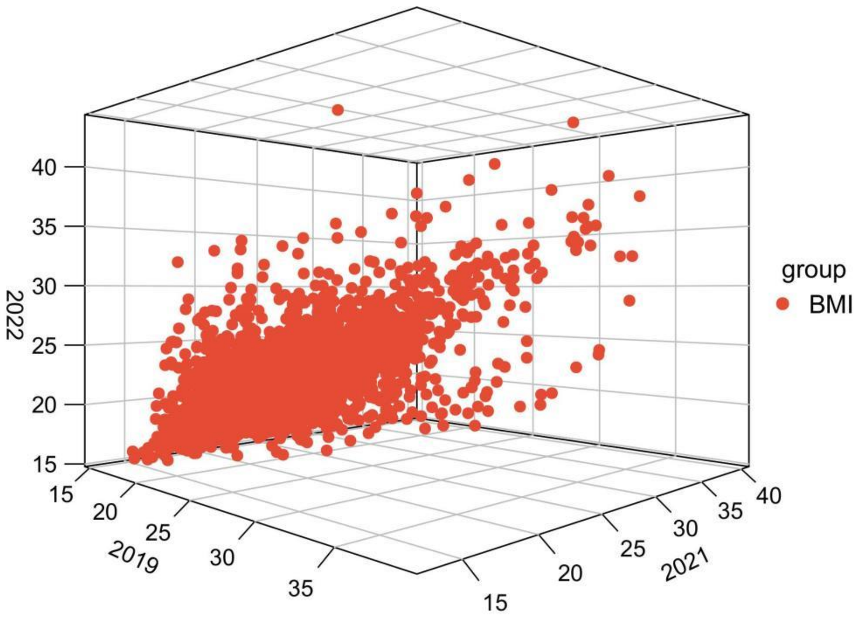

Figure 2. Three-dimensional correlation of BMI in males in 2019, 2021, and 2022

During 2019-2022, the annual growth rates of the obesity detection rate among the males were -1.00%, 2.49%, and 3.14% between adjacent years, respectively, and the figures for the females were -0.03%, 0.78%, and 0.81% between adjacent years, respectively. The annual growth rates of the obesity detection rate of both the males and the females declined slightly from 2019 to 2020, and then increased steadily in 2020-2022, and the annual growth rates of the males were much higher than those of the females.

3.3.2 Detection of overweight

In the longitudinal study on the university students' overweight detection rates, the overweight detection rate of the male university students increased from 19.98% in

2019 to 20.9% in 2022, and that of the female university students increased from 10.98% in 2019 to 11.85% in 2022. The figure for the entire population increased from 13.43% to 14.32%. In terms of gender stratification, the results of χ^2 test showed that the difference in overweight detection rates between male and female university students each year was statistically significant (P<0.01), with the males having a higher overweight detection rate than the females. It is worth noting that there was no significant increase in the overweight detection rate in the male and female groups from 2020, which was the first year of the COVID-19 pandemic. The overweight detection rate showed a significant decrease in 2021 and a significant increase in 2022 (Table 4).

Table 4. Comparison of overweight detection rates

	2019			2020			2021			2022			χ^2(trend)
Groups	Statistical value	Total number of pe	Number of detections (det	Statistical value	Total number of pe	Number of detections (det	Statistical value	Total number of pe	Number of detections (det	Statistical value	Total number of pe	Number of detections (det	

	ople	ection rate)	ople	ection rate)	ople	ection rate)	ople	ection rate)	
Male	1206	241 (19.98%)	1206	241 (19.8%)	1206	216 (17.91%)	1206	252 (20.9%)	0.897
Female	3207	352 (10.98%)	3207	334 (10.41%)	3207	299 (9.32%)	3207	380 (11.85%)	0.537
	χ^2	61.131**	χ^2	120.793**	χ^2	62.694**	χ^2	58.454**	
the total	4413	593 (13.43%)	4413	575 (13.03%)	4413	515 (11.67%)	4413	632 (14.32%)	0.57

Note: **P<0.01

Between 2019 and 2022, the annual growth rates of the overweight detection rate among the males were -0.18%, -1.89%, and 2.99% between adjacent years, respectively, and the figures for the females were -0.57%, -1.09%, and 2.53% between adjacent years, respectively. The annual growth rates of the overweight detection rate of both the males and the females showed a decreasing trend from 2019 to 2021, and continued to increase from 2021 to 2022, with the annual growth rate of the males being much higher than that of the females.

4 Conclusions

The results of this study show that (1) the physical fitness and health status of the university students enrolled in 2019 were seriously affected by COVID-19. In terms of body shape, the obesity detection rate increased year by year, and the overweight and obesity detection rates of the male students were even worse than those of the female students. (2) In terms of physical fitness, the flexibility of the university students enrolled in 2019 had been declining year by year under the influence of COVID-19; the endurance was basically unchanged in the first two years, and began to decline in the junior and senior stages; and the strength did not show a downward trend under the influence of the pandemic. We suggest that (1) school policies and physical education teachers should pay more attention to controlling the BMI of all adolescents and reducing the overweight and obesity detection rates, especially for the male university students, and that (2) focus should be placed on the ability of university students to

develop agility, explosiveness, and muscular strength at each stage, (3) and on improving cardiorespiratory endurance in the third and fourth years of university students. This study provides new insights for future explorations of the effects of school closures or online learning on the physical fitness of children and adolescents, and the results are expected to support the development of countermeasures aimed at enhancing adolescent health by government authorities and policy makers.

5 Data availability

Data is provided within the manuscript or supplementary information files.

6 Discussions

This study is the first longitudinal study to investigate the changes in physical fitness due to the impact of the COVID-19 pandemic during the four years of university for freshmen students enrolled in 2019 (before COVID-19). The study was aimed to investigate the longitudinal changes in the physical fitness of Chinese university students before and after the COVID-19 pandemic (2019-2022), especially the trends in body shape, physical function, physical fitness, and the detection rates of overweight and obesity. By analyzing the university students' physical fitness data over a four-year period, we aimed to reveal the impact of the pandemic on university students' health and provide a basis for future health interventions. The results of this study showed that the female university students exhibited significant temporal differences in height, weight, BMI, and other body shape indicators, while the male university students also

showed significant changes in weight and BMI, with no significant temporal differences in height indicators. In addition, the male and female students also showed significant changes in physical function and physical fitness indicators such as vital capacity, long jump, and sit-reach during the four-year period; the female students showed significant changes in 50-m run and sit-ups, while the male students did not show significant changes in 50-m-run and pull-ups. Obesity detection rates showed an increasing trend year by year among both the males and the females, especially during the severe pandemic in 2020, when obesity detection rates increased significantly. Obesity and overweight detection rates were much higher among the males than the females. Overweight detection rates among both male and female students showed a decreasing trend from 2019 to 2021, with significantly higher detection rates in 2022 than in the previous three years.

The results on changes in the body shape have similarities with the findings of a number of international studies that have documented the negative impact of the COVID-19 pandemic on the body shape. For example, a study by Aghababian, A. H. (2021) found that weight gain and obesity rates increased among adults during the COVID-19 pandemic, especially during lockdowns, with changes in eating habits being the main cause. The study found that the duration of binge eating episodes increased from 3.26 ± 1.89 hours to 3.92 ± 2.08 hours [18]. Hu et al. (2022) similarly found an increase in BMI and a decrease in physical fitness among university students following the COVID-19 lockdown due to prolonged social distancing and school

closures [19]. Although the COVID-19 pandemic appeared to be receding in 2022, the obesity and metabolic dysregulation pandemic continues to rage [20]. These are consistent with the fact that weight, BMI, and obesity rates among university students in this study showed a yearly trend over time. In addition, the present study also found that, between genders, the detection rates of overweight and obesity were much greater among the male students than among the female students during the four years of university, a finding that is also consistent with the findings of most previous studies [16, 21]. This phenomenon may be related to the fact that females are more body-conscious and therefore less likely to gain weight during the pandemic than males [16]. However, it is worth noting that a previous study noted a significant increase in adolescent obesity detection rates in 2020 compared to 2019, with a decreasing trend in 2021 and 2022 [22]. The reason for this discrepancy may be related to the age range of the sample size. The respondents of this study were university students with an average age of 18-20 years. The lack of teacher supervision and insufficient self-discipline may have led to an increase in the obesity detection rate year after year. Previous studies have focused on the adolescent population aged 6-14 years, and their conclusions may differ from those from the university students. In addition, the results of this study showed that the overweight detection rate among the university students showed a decreasing trend from 2019 to 2021 and was significantly higher in 2022 than in the previous three years. This finding differs from the results of a previous study that found an increasing trend in overweight detection rates among university students from 2019

to 2021 [23]. This difference may be related to sample selection. The previous study focused on data from 10 universities in Hunan Province, whereas this study selected only one teacher training university in Hunan Province, and the number of female students in that university was much higher than that of male students, with a large difference in the gender ratio of the samples, which may be one of the reasons for the inconsistent results.

Regarding the results of changes in physical fitness, this study fully discussed from three perspectives: cardiorespiratory fitness, muscular strength and flexibility. From the perspective of cardiorespiratory fitness, the scores of both genders from the 1,000-m run (boys) and 800-m run (girls) were significantly better in 2019 and 2020 than in 2021 and 2022. This change may be related to the characteristics of China's education system. In China’s higher education system, freshmen and sophomores are required to complete a certain number of physical education credits, whereas juniors and seniors are no longer subjected to a mandatory requirement for physical education [24]. In addition, physical activity (PA) and physical fitness (PF) levels of university students decline with age, with PF increasing from first to second year but gradually slowing from third to fourth year [25]. This finding is consistent with the results of a previous study, which noted a decreasing trend in endurance quality among university students one year after the implementation of COVID-19 restrictions (2020) [26]. From the perspective of muscular strength, the results of this study showed that the male students' performance in the 50-m run and pull-ups did not change significantly over

the four-year period, suggesting that male students' explosive and upper body strength remained stable during the COVID-19 pandemic. In contrast, the female students' performance in the 50-m run was significantly better in 2022 than in the previous three years, and their performance in 1-min sit-ups showed a year-to-year increase. In addition, long-jump scores were significantly lower in freshmen and seniors than in sophomores and juniors. These results suggest that muscle strength did not significantly decrease in both the males and the females as a result of COVID-19. This phenomenon may be attributed to the fact that during home quarantine at the beginning of the pandemic, the university's physical education program required students to perform daily resistance training, including coursework such as push-ups, sit-ups, and weighted squats. Although the course was conducted online, it was still supervised online by a teacher. In addition, in China's higher education system, coursework accounts for 20% of the score for physical education classes and counts toward a student's grade point average (GPA) [24]. This system may have motivated students to maintain a certain amount of exercise during the pandemic, thus maintaining muscle strength levels. From the perspective of flexibility, this study found a decreasing trend in flexibility indicators over time for both the male and female students. This result is consistent with previous studies suggesting that home quarantine during a pandemic led to a decrease in flexibility among university students [15, 27]. For example, a study by Chtourou et al. (2020) identified the lack of systematic stretching training during the pandemic as a major cause of decreased flexibility [28]. In addition, the limited range

of daily activities during home quarantine may have led to decreased joint mobility and muscle stretching, especially the iliopsoas muscle tightness of students due to prolonged sedentary periods resulting from online courses [29].

This study is subject to some limitations, although it study provides valuable data on the impact of the COVID-19 pandemic on the physical fitness of university students. First, the samples of this study were university students in a certain region of China, which may not fully represent the overall situation of university students nationwide. Second, the study data mainly relied on school physical examination data, which may have some measurement errors. In addition, this study failed to explore in depth the influence of psychological factors on physical fitness during the pandemic, and future studies could further explore the relationship between mental health and physical fitness. Based on the limitations of this study, future research can be conducted in the following ways: first, expand the sample size to cover more regions and different types of colleges and universities, so as to improve the representativeness and generalizability of the study. Second, more accurate measurement tools, such as accelerometers and body composition analyzers, can be used to reduce measurement errors. Finally, future studies could further explore the relationship between mental health and physical fitness during the pandemic, especially the effects of psychological factors such as anxiety and depression on body weight and physical fitness.

The findings of this study have important practical implications for public health and health promotion. The findings emphasize the importance of maintaining a healthy lifestyle during a pandemic, especially in terms of diet and physical activity. Second, the findings suggest the need for educational institutions and public health authorities to develop targeted interventions to address the growing of obesity in the university students. Finally, this study provides a scientific basis for future health policy development, especially on how to balance pandemic prevention and control with health promotion.

7 Author Contributions

Conceptualization, L.G. and L.J.; methodology, H.H.; software, H.H.; validation, H.H., L.G. and L.J.; formal analysis, L.G.; investigation, L.J.; resources, L.J.; data curation, H.H.; writing—original draft preparation, L.G.; writing—review and editing, L.J.; visualization, H.H.; supervision, L.J.; project administration, L.J.; funding acquisition, L.J. All authors have read and agreed to the published version of the manuscript.

8 Funding

This research was funded by the Research Project on Teaching Reform of General Colleges and Universities in Hunan Province in 2023 (Project No. HNJG-20230872), the Fourteenth Five-Year Plan of Educational Science in Hunan Province in 2024 (Project No. XJK24CTW013), and the Deepening Educational Evaluation Reform in the New Era in Hunan Province in 2022 Pilot Project (Project No. TGYX202207).

9 Conflict of Interest

The authors declare that the research was conducted in the absence of any commercial or financial relationships that could be construed as a potential conflict of interest.

10 Acknowledgments

We are very grateful to all the students who participated in the testing.

11 Institutional Review Board Statement

The study was conducted in accordance with the Declaration of Helsinki, and approved by the Institutional Review Board of the School of Sports Science, Hengyang Normal University OF INSTITUTE(September 18, 2019).

12 Informed Consent Statement

Informed consent was obtained from all subjects involved in the study.

13 References

1. Chinazzi M, Davis JT, Ajelli M, Gioannini C, Litvinova M, Merler S, et al. The effect of travel restrictions on the spread of the 2019 novel coronavirus (COVID-19) outbreak. Science. 2020;368(6489):395-400.
2. Ammar A, Brach M, Trabelsi K, Chtourou H, Boukhris O, Masmoudi L, et al. Effects of COVID-19 home confinement on eating behaviour and physical activity: results of the ECLB-COVID19 international online survey. 2020;12(6):1583.
3. Niedermeier M, Frühauf A, Kopp-Wilfling P, Rumpold G, Kopp M. Alcohol Consumption and Physical Activity in Austrian College Students—A Cross-Sectional Study. Substance Use & Misuse. 2018;53(10):1581-90.
4. López-Bueno R, López-Sánchez GF, Casajús JA, Calatayud J, Tully MA, Smith L. Potential health-related behaviors for pre-school and school-aged children during COVID-19 lockdown: A narrative review. Preventive Medicine. 2021;143.
5. Wanner M, Richard A, Martin B, Faeh D, Rohrmann S. Associations between self-reported and objectively measured physical activity, sedentary behavior and overweight/obesity in NHANES 2003–2006. International Journal of Obesity. 2016;41(1):186-93.

6. Moore SC, Lee IM, Weiderpass E, Campbell PT, Sampson JN, Kitahara CM, et al. Association of Leisure-Time Physical Activity With Risk of 26 Types of Cancer in 1.44 Million Adults. JAMA Internal Medicine. 2016;176(6).
7. Aune D, Norat T, Leitzmann M, Tonstad S, Vatten LJ. Physical activity and the risk of type 2 diabetes: a systematic review and dose–response meta-analysis. European Journal of Epidemiology. 2015;30(7):529-42.
8. Deng C-H, Wang J-Q, Zhu L-M, Liu H-W, Guo Y, Peng X-H, et al. Association of Web-Based Physical Education With Mental Health of College Students in Wuhan During the COVID-19 Outbreak: Cross-Sectional Survey Study. Journal of Medical Internet Research. 2020;22(10).
9. Hamdani SMZH, Zhuang J, Hadier SG, Khurram H, Hamdani SDH, Danish SS, et al. Establishment of health related physical fitness evaluation system for school adolescents aged 12–16 in Pakistan: a cross-sectional study. Frontiers in Public Health. 2023;11.
10. Basterfield L, Burn NL, Galna B, Batten H, Goffe L, Karoblyte G, et al. Changes in children's physical fitness, BMI and health-related quality of life after the first 2020 COVID-19 lockdown in England: A longitudinal study. Journal of Sports Sciences. 2022;40(10):1088-96.
11. Pinho CS, Caria ACI, Aras Júnior R, Pitanga FJG. The effects of the COVID-19 pandemic on levels of physical fitness. Revista da Associação Médica Brasileira. 2020;66(suppl 2):34-7.
12. Béghin L, Thivel D, Baudelet J-B, Deschamps T, Ovigneur H, Vanhelst J. Change in physical fitness due to the COVID-19 pandemic lockdown in French adolescents: a comparison between two independent large samples from Diagnoform battery. European Journal of Pediatrics. 2022;181(11):3955-63.
13. Li H, Cheong JPG. The Impact of the COVID-19 Pandemic on the Physical Fitness of Primary School Students in China Based on the Bronfenbrenner Ecological Theory. Frontiers in Psychology. 2022;13.
14. Ganzar LA, Salvo D, Burford K, Zhang Y, Kohl HW, Hoelscher DM. Longitudinal changes in objectively-measured physical activity and sedentary time among school-age children in Central Texas, US during the COVID-19 pandemic. International Journal of Behavioral Nutrition and Physical Activity. 2022;19(1).
15. Zhai X, Sawada SS, Shi M, Xiong C, Koriyama S, Dimitroff SA, et al. Impact of COVID-19 Pandemic Lockdown on Body Mass Index and Physical Fitness in Chinese College Students. The Journal of adolescent health : official publication of the Society for Adolescent Medicine. 2024;74(5):892-9.
16. Li H, Song Y, Wang Y, Feng X, Li C, Peng J, et al. Impact of the COVID-19 pandemic lockdown on Body Mass Index: a three-year follow up study in 6,156 Chinese college students. Frontiers in Endocrinology. 2024;15.
17. Chinese Student Physique and Health Research Group.Report on the physical fitness and health surveillance of Chinese school students in 2019. Beijing: Higher Education Press; 2019.
18. Aghababian AH, Sadler JR, Jansen E, Thapaliya G, Smith KR, Carnell S. Binge Watching during COVID-19: Associations with Stress and Body Weight. Nutrients. 2021;13(10).
19. Hu D, Liu Z, Zhang H, Jiang C. Changes in Physical Health-Related Indexes of Chinese College Students before and after COVID-19 Lockdown. Biomed Res Int. 2022;2022:7802492.
20. Boutari C, Mantzoros CSJM. A 2022 update on the epidemiology of obesity and a call to action: as its twin COVID-19 pandemic appears to be receding, the obesity and dysmetabolism pandemic continues to rage on. Elsevier; 2022. p. 155217.
21. Midulla F, Cimino G, Nenna R, Troiani P, D'Alù V, Cappelli C, et al. COVID-19 Lockdown Impacts Among Patients with Cystic Fibrosis: An Italian Regional Reference Centre Experience. Frontiers in Bioscience-Landmark. 2022;27(6).
22. Qin G, Li H, Qin Y, Liu B. Is the COVID-19 epidemic affecting the body mass of Chinese teenagers? – A longitudinal follow-up study. Frontiers in Public Health. 2023;11.
23. Jiang Q, Huang X, Wang Z, Dai X, Li R, Cui DJFiPH. Regional differences of

physical fitness and overweight and obesity prevalence among college students before and after COVID-19 pandemic since the “double first-class” initiative in China. 2024;11:1252270.
24. Circular of the Ministry of Education on Printing and Distributing the Basic Standards for Physical Education in Institutions of Higher Learning. 2014 [Available from: http://www.moe.gov.cn/srcsite/A17/moe_938/s3273/201406/t20140612_171180.html.
25. Wang J. The association between physical fitness and physical activity among Chinese college students. Journal of American College Health. 2019;67(6):602-9.
26. Yu H, An S, Tao Y, Austin L. Correlation and Change in Physical Activity and Physical Fitness across Four Years of College Students after One Year of COVID-19 Lockdown. Healthcare (Basel). 2022;10(9).
27. Jie C, Xugui S, Min Z, Ergang Z, Hongwu W, Jun S. The influence of home isolation during COVID-19 on the physical fitness development of college students: a study utilizing repeated measures analysis of variance. BMC Public Health. 2023;23(1):2186.
28. Chtourou H, Trabelsi K, H'Mida C, Boukhris O, Glenn JM, Brach M, et al. Staying Physically Active During the Quarantine and Self-Isolation Period for Controlling and Mitigating the COVID-19 Pandemic: A Systematic Overview of the Literature. Frontiers in Psychology. 2020;11.
29. Goyal N, Chaudhary V. Effect of BMI on Tightness of Iliopsoas Muscle in Students due to Prolonged Sitting for Online Classes During COVID-19 Pandemic. Journal of Clinical and Diagnostic Research. 2022.

Click here to access/download
Supporting Information
Supplementary Material.docx

Click here to access/download
Supporting Information
Ethical approval(2).pdf
