# Supplementary material for: A longitudinal study of four-year changes in physical fitness among university students before and after COVID-19: 2019–2022
Source: PLoS One. 2025 Oct 10;20(10):e0334088. doi: 10.1371/journal.pone.0334088 (PMC12513638; doi:10.1371/journal.pone.0334088)
Supplement: Attachment — Submitted filename: Response to Reviewers.docx [file pone.0334088.s004.docx]

Dear Editors and reviewers

On behalf of all the contributing authors, I would like to express our sincere appreciations of your letter and reviewers' constructive comments concerning our article entitled “A longitudinal study of four-year changes in physical fitness among university students before and after COVID-19: 2019-2022” (Manuscript No:PONE-D-25-29142R1). These comments are all valuable and helpful for improving our article. Based on the comments of the editor-in-chief and reviewers, we made extensive revisions to the manuscript.In this revised version. changes to our manuscript were all highlighted within the document by using red-colored text.Point-by point responses to the nice editor and five nice reviewers are listed below this letter.

**Point-by point responses**

**Academic editor:**

Journal Requirements:

When submitting your revision, we need you to address these additional requirements.

1.Please ensure that your manuscript meets PLOS ONE’s style requirements, including those for file naming. The PLOS ONE style templates can be found at

https://journals.plos.org/plosone/s/file?id=wjVg/PLOSOne_formatting_sample_main_body.pdf and

<https://journals.plos.org/plosone/s/file?id=ba62/PLOSOne_formatting_sample_title_authors_affiliations.pdf>

**The author’s answer:**

We confirm that the revised manuscript has been carefully formatted to meet all PLOS ONE style requirements. Specifically, we have: (1) adjusted the document structure to align with the journal’s template (e.g., title page, headings, reference style); (2) standardized file naming conventions as specified; and (3) ensured consistency in font, spacing, and section organization per the provided guidelines (https://journals.plos.org/plosone/s/file?id=wjVg/PLOSOne_formatting_sample_main_body.pdf and https://journals.plos.org/plosone/s/file?id=ba62/PLOSOne _formatting_sample_title_authors_affiliations.pdf). All changes have been verified to comply fully with the journal’s formatting policies.

**Academic editor:**

2. We note that the grant information you provided in the ‘Funding Information’ and ‘Financial Disclosure’ sections do not match.

When you resubmit, please ensure that you provide the correct grant numbers for the awards you received for your study in the ‘Funding Information’ section.

**The author’s answer:**

We sincerely apologize for the oversight in the initial submission. We have now carefully verified and corrected the grant information in both the ‘Funding Information’ and ‘Financial Disclosure’ sections to ensure complete consistency. The accurate grant numbers and funding details have been updated in the manuscript.

**Academic editor:**

3.Thank you for stating the following financial disclosure:

“This research was funded by the Research Project on Teaching Reform of General Colleges and Universities in Hunan Province in 2023 (Project No. HNJG-20230872), the Fourteenth Five-Year Plan of Educational Science in Hunan Province in 2024 (Project No. XJK24CTW013), and the Deepening Educational Evaluation Reform in the New Era in Hunan Province in 2022 Pilot Project (Project No. TGYX202207).”

Please state what role the funders took in the study.  If the funders had no role, please state: "The funders had no role in study design, data collection and analysis, decision to publish, or preparation of the manuscript."

If this statement is not correct you must amend it as needed.

Please include this amended Role of Funder statement in your cover letter; we will change the online submission form on your behalf.

**The author’s answer:**

The revised funder role statement has been indicated in red font in the cover letter.

**Academic editor:**

4. Please note that funding information should not appear in any section or other areas of your manuscript. We will only publish funding information present in the Funding Statement section of the online submission form. Please remove any funding-related text from the manuscript.

**The author’s answer:**

All expressions about the fund have been removed from the manuscript.

**Academic editor:**

5. PLOS requires an ORCID iD for the corresponding author in Editorial Manager on papers submitted after December 6th, 2016. Please ensure that you have an ORCID iD and that it is validated in Editorial Manager. To do this, go to ‘Update my Information’ (in the upper left-hand corner of the main menu), and click on the Fetch/Validate link next to the ORCID field. This will take you to the ORCID site and allow you to create a new iD or authenticate a pre-existing iD in Editorial Manager.

**The author’s answer:**

ORCID iDs have been validated in Editorial Manager.

**Academic editor:**

6. Your ethics statement should only appear in the Methods section of your manuscript. If your ethics statement is written in any section besides the Methods, please move it to the Methods section and delete it from any other section. Please ensure that your ethics statement is included in your manuscript, as the ethics statement entered into the online submission form will not be published alongside your manuscript.

**The author’s answer:**

The ethics statement is adjusted in the method section of the manuscript and removed from any other section.

**Academic editor:**

7. Please remove all personal information, ensure that the data shared are in accordance with participant consent, and re-upload a fully anonymized data set.

Note: spreadsheet columns with personal information must be removed and not hidden as all hidden columns will appear in the published file.

Additional guidance on preparing raw data for publication can be found in our Data Policy (https://journals.plos.org/plosone/s/data-availability#loc-human-research-participant-data-and-other-sensitive-data) and in the following article: <http://www.bmj.com/content/340/bmj.c181.long>.

**The author’s answer:**

All personal information has been deleted to ensure that the data shared meets the consent of the participants.

**Academic editor:**

8. Please include captions for your Supporting Information files at the end of your manuscript, and update any in-text citations to match accordingly. Please see our Supporting Information guidelines for more information: http://journals.plos.org/plosone/s/supporting-information.

**The author’s answer:**

A title for the supporting information file has been added to the end of the manuscript and any text citations have been updated accordingly to match.

9.If the reviewer comments include a recommendation to cite specific previously published works, please review and evaluate these publications to determine whether they are relevant and should be cited. There is no requirement to cite these works unless the editor has indicated otherwise.

**Academic editor:**

We carefully evaluated the four references provided by the reviewers. Three of these are new documents [Ref 8, 19, 27] and one is a citation that we have already incorporated [Ref 26].

**Point-by point responses**

**Reviewer 1：**

The manuscript presents original research that investigates changes in body composition and physical fitness among Chinese university students over a four-year period spanning before, during, and after the COVID-19 pandemic. The topic is of public health relevance, and the authors have collected a large dataset with multiple fitness indicators. Overall, the study meets the journal’s criteria for technical rigor and ethical compliance. However, several aspects require minor revisions to improve the clarity, consistency, and presentation of the work.

1. Scientific Rigor and Methods:

The cohort design is appropriate for the research objectives, and the repeated physical fitness testing across four years is a strength.

The statistical approach is acceptable for basic trend analysis. Nevertheless, the rationale for selecting ANOVA over more robust longitudinal models could be briefly explained. Additionally, more detail on data collection quality control (e.g., measurement consistency) is encouraged.

**The author’s answer:**

We appreciate the reviewer’s constructive feedback. The rationale for using ANOVA has been clarified, emphasizing its suitability for comparing adjacent years while addressing within-group variability. Additionally, details on quality control measures (e.g., standardized testing procedures and calibrated equipment) have been added to strengthen the methodological rigor. These revisions enhance the transparency and robustness of the statistical analysis.

**Reviewer 1：**

2. Tables and Figures:

Tables 2, 3, and 4 require formatting adjustments for clarity. Some rows and columns are misaligned, and headers are difficult to follow. Similarly, Figures 1 and 2 do not effectively communicate the temporal changes in BMI. Consider replacing the 3D scatterplots with simpler and more interpretable line charts or boxplots.

**The author’s answer:**

We appreciate the reviewer’s valuable suggestions regarding the presentation of tables and figures. In response, we have reformatted Tables 2, 3, and 4 to ensure proper alignment and improve header clarity for better readability.

In response to “reviewer 3” feedback, we have retained the original “3D scatterplots,” which were praised for their effective visualization of multidimensional relationships. To further improve interpretability, we have supplemented these with "boxplots" to better highlight temporal trends in BMI distribution. The "Results" section has been updated to incorporate both visualization methods, ensuring a more comprehensive and accessible presentation of the data. These modifications aim to balance innovative graphical representation with clear communication of distributional trends over time.

**Reviewer 1：**

3. Discussion Section:

The manuscript lacks a focused discussion section. Beyond reporting results, the paper should include critical interpretation, explanations for observed trends, comparison with existing studies, and acknowledgment of limitations. A structured discussion would greatly improve the manuscript’s clarity and depth.

**The author’s answer:**

We sincerely appreciate the reviewer’s constructive feedback regarding the need for a more structured and in-depth discussion. In response, we have thoroughly revised the Discussion section to provide a critical interpretation of the findings, including potential explanations for the observed trends in overweight and obesity rates. We have also incorporated comparisons with relevant existing studies to contextualize our results and added a clear acknowledgment of the study’s limitations, such as potential confounding factors and measurement constraints. These enhancements aim to improve the manuscript’s clarity, depth, and scholarly contribution. Specific modifications have been marked in red in the discussion section.

**Reviewer 1：**

4. Writing and Organization:

The manuscript is generally well written in standard English. Minor language polishing could improve clarity and readability. The structure would benefit from clearer section transitions and concise topic sentences in the results and discussion.

**The author’s answer:**

We have performed language polishing throughout the manuscript to enhance readability and precision. Additionally, we have improved section transitions and refined topic sentences in the Results and Discussion to ensure clearer logical progression. As suggested, we have also incorporated subheadings in the Discussion section to better structure the interpretation of findings.

**Reviewer 1：**

5. Ethics and Data Availability:

Ethical approval and informed consent are clearly stated.

Data availability is appropriately addressed and aligns with PLOS ONE standards.

Recommendation:

The study is scientifically sound and methodologically appropriate. With minor revisions focused on improving table formatting, figure clarity, and manuscript organization, it will be suitable for publication.

**The author’s answer:**

We sincerely appreciate the reviewer's positive assessment of our study's scientific validity and ethical compliance. In accordance with the recommendations, we have carefully revised the Ethics and Data Availability section to fully align with PLOS ONE standards, ensuring explicit transparency regarding ethical approval, participant consent, and data accessibility.

**Reviewer 2：**

Dear Authors,

Congratulations on your choice of topic. I read the article with interest. The manuscript provides important information on the physical fitness of students in the context of the COVID-19 pandemic. The study is based on measurements of physical fitness (tests), body weight and height, and spirometry. The description lacks information on whether the researchers conducting the study had been trained beforehand. The paper also does not indicate whether the study was conducted using uniform, calibrated measuring devices, which seems important from the point of view of the comparability of the results. The study has its limitations, but the authors have drawn attention to them by describing them in the limitations section. In their recommendations for the future, they suggest, among other things, the use of objective tools, such as accelerometers, which will allow for a more precise and reliable assessment of physical activity levels.

The study focuses on a large group of students from Hunan Province, but it should be noted that this group is not representative of the entire student population. Although this sample structure provides valuable data, it may not fully reflect the specific cultural conditions and barriers characteristic of this group. The authors recognise this and draw attention to it when discussing the limitations of the study.
Detailed suggestions are included in the comments on the article.

**The author’s answer:**

We sincerely appreciate the reviewers' thoughtful comments and valuable suggestions that have significantly strengthened our manuscript. In direct response to the expert's recommendations, we have enhanced Section 2.1 by explicitly stating that: (1) all researchers underwent standardized training to ensure measurement consistency and protocol adherence, and (2) all physical fitness assessments were conducted using uniform, calibrated measuring devices to guarantee data comparability across testing periods. These important additions address the methodological concerns regarding measurement reliability and procedural standardization while maintaining the study's original framework. The modifications improve the transparency and scientific rigor of our methods section without altering the fundamental study design. We fully acknowledge the limitation regarding regional representativeness in our discussion section and have incorporated the expert's suggestion about future use of accelerometers in our recommendations for further research.

**Reviewer 3：**

General Comments

Thank you for the opportunity to review this article, and I would like to congratulate the authors on their efforts in compiling this work.

The study presents original research, a longitudinal analysis of physical fitness changes in Chinese university students before and after the COVID-19 pandemic (2019-2022). The research focus and dataset are novel, and the manuscript explicitly addresses a gap in the literature regarding long-term impacts of the pandemic on university students.

However, the manuscript would benefit from further adjustments and revisions as proposed herein, section by section.

Title and Short Title

• The titles are clear and accurately reflect the study's content.

• I suggest you ensure consistency in the use of “pre/post-COVID-19” versus "before and after COVID-19" if used in other materials (e.g., in databases or indexing).

**The author’s answer:**

We thank the reviewer for this important observation regarding terminology consistency. In response, we have standardized all temporal references to COVID-19 throughout the manuscript, figures, and supplementary materials to exclusively use “before and after COVID-19” as the preferred terms.

**Reviewer 3：**

Abstract

• As it is, the abstract is too long. The number of words is almost double the recommended standard by PLOS ONE. Consider slightly rephrasing some sentences for tighter prose and conciseness, typical for abstracts where every word counts.

• Be attentive to the use of capitalization; for instance, in line 28, the letter 'V' in the word "vital" should be capitalized. Review the entire document for any similar inconsistencies.

• Consider providing keywords at the end of the abstract to improve indexing and discoverability in academic databases and search engines.

**The author’s answer:**

We sincerely appreciate the reviewers' constructive feedback and have carefully addressed all suggestions: (1) we have substantially shortened the abstract (now 236 words) through tighter phrasing and removal of redundant information while preserving key findings; (2) all capitalization errors (including “Vital”) have been corrected throughout the manuscript; and (3) we have added 5 relevant keywords (COVID-19; physical fitness; body shape; university students; longitudinal study) at the end of the abstract to enhance discoverability.

**Reviewer 3：**

Introduction

• While the literature review effectively identifies the research gap, consider briefly strengthening the rationale for why a four-year longitudinal study from 2019 (pre-pandemic) to 2022 (post-pandemic) is especially valuable compared to existing shorter-term studies. What specific insights does this longer timeframe offer that others do not?

• Where some words have been defined in the first appearance and an abbreviation provided, the use of the abbreviation in the succeeding sections is best practice, e.g., physical activity (PA). Use PA in the succeeding paragraphs—see lines 66, 69, 79… Scan through the entire manuscript and use the abbreviations where applicable.

• Revise the use of tense in line 65.

• Is this study grounded on any theory that support its scientific constructs?

**The author’s answer:**

We appreciate the reviewers’ constructive comments and have carefully addressed each point: (1) The rationale for the four-year longitudinal design (2019-2022) has been strengthened by emphasizing its unique capacity to capture pre-pandemic baselines, acute-phase declines during lockdowns, and post-lockdown recovery patterns - providing insights into long-term trends that shorter studies cannot discern; (2) We have systematically implemented abbreviations (e.g., PA for physical activity) throughout the manuscript as recommended, ensuring consistency in subsequent mentions after initial definitions; (3) The tense in line 65 has been revised to past tense (“led”) to accurately reflect completed pandemic-related impacts; (4) Regarding theoretical grounding, while this study primarily employs an empirical longitudinal approach to track fitness trends, we acknowledge that future research could benefit from incorporating theoretical frameworks to further explain observed behavioral and physiological changes. We have added discussion of potential theoretical applications (e.g., ecological models) in the limitations section to address this consideration.

**Reviewer 3：**

Materials and Methods

While the methods are generally described, more details should be provided for full reproducibility. Consider addressing the following:

• How were the participants selected, and what criteria were used to ensure a representative sample? Were there any inclusion or exclusion criteria (e.g., pre-existing health conditions, baseline fitness levels)?

**The author’s answer:**

Modifications made: The Methods section was revised to clarify participant selection by adding explicit inclusion/exclusion criteria. All first-year students were eligible (inclusion), while those with chronic health conditions (e.g., cardiovascular disease) or physical disabilities impairing exercise participation were excluded. Representativeness was addressed by enrolling all consenting freshmen from the 2019 cohort at the Hunan teacher training university, reducing selection bias.

**Reviewer 3：**

• Did the physical education professors receive any specific training to ensure uniform test administration? Were the same professors involved across the four years?

**The author’s answer:**

The original sentence was revised to specifically address: the continuity of the assessment team across the study duration. (2.1 Study population)

**Reviewer 3：**

• Detail in brief the specific warm-up routines in line 118.

**The author’s answer:**

The Methods section (2.2 Physical Fitness Test) was revised to include specific warm-up protocols to enhance methodological transparency.

**Reviewer 3：**

• Were inter-rater reliability checks performed for any of the subjective measurements, for example, the form for sit-ups, long jump, or sit-reach?

• Were there any potential variations in the testing environment (e.g., indoor vs. outdoor, time of day, equipment type) across the four years that could influence results? If so, how were these controlled or accounted for in the analysis or discussion?

• How was consistent participant engagement and adherence to test protocols ensured over the four years of data collection?

**The author’s answer:**

Added explicit mention of standardized training and scoring criteria for all subjective assessments to ensure measurement consistency across testers.

Specified that all tests were conducted in indoor venues using standardized equipment.

Clarified that testing occurred during consistent morning sessions to minimize diurnal variation.

Added that any potential variations were documented and accounted for statistically.

The revised text now appears in section 2.2 as shown in the updated manuscript. We believe these changes substantially improve the methodological rigor and clarity of our physical fitness testing protocol.

**Reviewer 3：**

• In line 170, I am not sure the correct symbol for interpretation has been used. Should this remain as 24 ≤ BMI < 27.9 or ≥ 24 BMI < 27.9? The former would mean even a BMI of 20 kg/m² was still ranked as overweight.

**The author’s answer:**

Thanks to the reviewer for reminding us that we have verified that there is no problem with this statement.

**Reviewer 3：**

• How was missing data handled, especially for students who dropped out or missed annual tests? Could you report the retention rate and analyze differences between completers and dropouts to address potential attrition bias?

**The author’s answer:**

In this study, all analyzed students strictly completed the full four-year testing cycle, and those who missed annual tests (including dropouts) were excluded from the final dataset per the research design. We acknowledge the importance of tracking attrition patterns in longitudinal research to assess potential bias, and this has been noted as an area for improvement in future studies. While our current analysis focuses on completers to ensure data consistency, we recognize that examining differences between completers and dropouts could further strengthen validity, and we will incorporate such analyses in subsequent work.

**Reviewer 3：**

• Important—Provide more details on the specifications (type, model number, etc.), calibration, and validation of the equipment used for physical fitness screening for physical fitness tests to ensure measurement accuracy.

**The author’s answer:**

All physical fitness measurements were conducted using standardized, calibrated equipment to ensure accuracy: Vital capacity was assessed using the GMCS-IV digital spirometer (Jianmin, Beijing, China; range: 0-10 L, resolution: 10 mL, ATS/ERS-compliant), while sit-and-reach flexibility was measured with the GMCS-IV flexibility tester (Jianmin, Beijing, China; range: 0–50 cm, resolution: 0.1 cm). Both devices were factory-calibrated and underwent daily verification using certified calibration tools (3.0 L syringe for spirometry, mechanical block for flexibility tester). Measurement protocols followed manufacturer guidelines and international standards (ATS/ERS for spirometry, ACSM for flexibility testing), with triplicate measurements taken and the best value recorded.

**Reviewer 3：**

• Provide a brief justification for the use of gender-specific fitness tests (e.g., pull-ups for males and sit-ups for females) and explain the rationale behind these choices.

**The author’s answer:**

The original text was modified by adding a brief justification for the gender-specific tests. The explanation highlights that pull-ups and the 1,000-m run for boys assess upper-body strength and endurance, while sit-ups and the 800-m run for girls evaluate core strength and aerobic capacity.（2.2 Physical fitness test）

**Reviewer 3：**

• Clarify whether adjustments were made for potential confounding variables (e.g., age, baseline fitness, socioeconomic status) in the statistical analysis.

**The author’s answer:**

The modified version explicitly acknowledges in the statistical analysis section that potential confounding variables (age, baseline fitness, socioeconomic status) were not adjusted for in the analysis, which enhances methodological transparency while maintaining all original analytical procedures and technical details intact.

**Reviewer 3：**

Results

Generally, the results section is comprehensive and well-structured, with clear presentation of longitudinal trends and gender differences. The use of visualizations (e.g., 3D scatterplots) is commendable. However, the following needs improvement:

• In line 194, is the 7 ≤ YEAR ≤ 19-year-old age group accurate, or did you imply 17 instead of 7? Clarify

• Within table 1, the use of the symbol is not clear to me. Should it be presented as 17≤year≤19 or 17≥year≤19? The use of less than and greater than symbols is confusing the interpretation and understanding of this age group. Please clarify.

**The author’s answer:**

Thank you for pointing this out. It was indeed a error—the correct lower limit should be 17 instead of 7. We have revised it to “17 ≤ YEAR ≤ 19” for accuracy.

Regarding the notation in Table 1:

The expression “17 ≤ year ≤ 19” is mathematically correct, indicating that the age group includes individuals aged 17 to 19 years old. We confirm that this notation is appropriate and have retained it in the revised manuscript.

**Reviewer 3：**

• In line 211, does the phrase “The same significant difference” imply similarly or that the mean weight of the female group equalled that of the male group?

• The authors need to adjust the font used in tables 1-4 to ensure proper alignment of text and values. As it is, they are juggled, making readability difficult.

• All the tables require proper formatting by improving row and column alignment to enhance readability. Tables 3 and 4 are very difficult to follow.

• Define abbreviations as a footnote where applicable in the tables, e.g., BMI.

**The author’s answer:**

Removed ambiguous phrasing. Replaced “The same significant difference” with “A significant difference” to avoid confusion.

We sincerely appreciate the reviewer’s constructive feedback. In response, we have carefully revised Tables 1–4 to improve readability and alignment. Specifically, we have adjusted the font style and spacing to ensure consistent text and value alignment. The row and column formatting has been standardized for clarity, with particular attention given to Tables 3 and 4 to enhance data presentation. Additionally, all abbreviations (e.g., BMI) have been defined in the table footnotes where applicable. These changes aim to ensure the tables are more accessible and easier to interpret.

**Reviewer 3：**

Discussion

• Discuss how pandemic-related disruptions (e.g., lockdowns, online learning) were accounted for in the analysis and whether any adjustments were made for reduced physical activity during these periods.

• Consider discussing the potential long-term implications of the observed physical fitness changes on health outcomes, such as risk for chronic diseases or mental health issues.

**The author’s answer:**

TRegarding pandemic-related disruptions, the analysis explicitly highlights the correlation between severe 2020 lockdowns, widespread online learning, and the significant surge in obesity rates, framing lockdown-induced restrictions on outdoor activities and reduced structured physical education under online learning as key contextual factors explaining the 2020 obesity increase.

For long-term health implications, the revision supplements discussions on potential lasting consequences of observed fitness declines (e.g., reduced cardiorespiratory fitness, decreased flexibility) and rising obesity rates。

**Reviewer 3：**

• Discuss the practical significance of statistically significant findings, especially for small changes in physical fitness indicators.

• Please make sure all abbreviations are presented in full in the first appearance in the manuscript, e.g., physical fitness has been mentioned from line 53 but only abbreviated from line 396.

• Consider adding a section on the strengths of the study before the limitations section.

• In the limitations part, consider also addressing potential biases inherent in a longitudinal study, such as selection bias due to dropouts or measurement bias if test administrators changed over time.

**The author’s answer:**

Regarding the practical significance of statistically significant findings, particularly for small changes in physical fitness indicators, I have expanded the relevant discussion to elaborate on how such findings can inform targeted interventions, monitor subtle progress in fitness programs, . All abbreviations have been carefully checked to ensure their full forms are provided upon first appearance in the manuscript. In response to the suggestion of adding a section on the strengths of the study before the limitations, and considering the overall length of the discussion section which required compression, I have adjusted the structure: the section on practical significance has been repositioned before the limitations. For the limitations section, I have supplemented it to address potential biases inherent in longitudinal studies, including selection bias arising from participant dropouts and measurement bias due to changes in test administrators over time.

**Reviewer 3：**

Conclusions

• The conclusions should be provided after the discussion section.

• The conclusions are presented appropriately and directly stem from the study's findings. If possible, consider summarizing the main limitations and future research directions in the conclusion to provide a balanced perspective.

**The author’s answer:**

In response to expert feedback, the revised conclusion streamlines language while retaining core findings, clearly linking COVID-19 impacts to specific fitness metrics (e.g., rising male obesity rates, declining flexibility, and late-stage endurance drops) and targeted recommendations. To enhance balance, it explicitly notes a key limitation—the focus on a single cohort—and proposes future directions, such as expanding to multi-cohort or longitudinal analyses to strengthen generalizability, thereby aligning with suggestions to contextualize results and guide further research.

**Reviewer 3：**

Presentation and Language

• There are some minor grammatical awkwardness and phrasings that could be improved for clarity and conciseness, e.g., line 331 “the study was aimed to” instead of “the study aimed to.” Scan the entire manuscript keenly for these kinds of errors.

• Proofread the entire manuscript thoroughly for minor spelling mistakes, e.g., recoded in line 155 instead of recorded.

• Ensure proper spacing where necessary, e.g., in line 185, space between the word rate, the equals sign, and the bracket.

• Some sentences are complex and could be simplified for broader accessibility.

**The author’s answer:**

We have carefully scanned the entire manuscript to address grammatical awkwardness and improve phrasing for enhanced clarity and conciseness; for instance, we corrected "the study was aimed to" in line 331 to "the study aimed to" as suggested. A thorough proofread was conducted to eliminate minor spelling mistakes, including revising "recoded" in line 155 to "recorded." We have also ensured proper spacing throughout the manuscript, such as adding the necessary space between the word "rate," the equals sign, and the bracket in line 185. Additionally, complex sentences have been simplified to improve broader accessibility, with careful attention paid to maintaining the accuracy and integrity of the content while enhancing readability. We believe these revisions have significantly improved the overall quality of the manuscript, and we hope they meet the expert's expectations.

**Reviewer 3：**

Data Availability Statement

• This statement should be provided after the discussion section.

• You state, "All relevant data are within the manuscript and its supporting information files." While this is generally acceptable, PLOS ONE strongly encourages data deposition in publicly accessible repositories. If possible, consider depositing your raw data in a recognized repository and providing the accession/version number in this section. If the data cannot be made fully public (e.g., due to privacy concerns), please provide a clear statement on how other researchers can request access to the data, including contact information or a data access committee.

**The author’s answer:**

Thanks to the reviewers for their suggestions to adjust the position of the Data Availability Statement.

**Reviewer 3：**

Ethics Statement

• As stated in lines 111 and 112, can the authors clarify why oral consent was used instead of written consent? Yet in the provided ethical approval, in the section on review of content, number 1, the board expressly states the following: “All participants will be required to sign an informed consent form, clearly understanding the purpose of the study, data collection methods, potential risks, and safeguards.”

**The author’s answer:**

Thanks to the reviewer’s suggestion, the ethics statement in the article has been revised according to the reviewer’s suggestion.

**Reviewer 3：**

References

• Ensure uniform in-text citations. There is a mix-up in some paragraphs. Refer to lines 354 and 358 against other paragraphs in the document and harmonize as necessary.

• Are you able to provide DOIs (Digital Object Identifiers) assigned to the cited journal papers where available? This ensures reliability and accuracy in citations.

• For online references, provide the date of accession and the link to the document.

**The author’s answer:**

We have carefully addressed the reviewer's suggestions to improve the manuscript. Regarding the first point, we have harmonized the in-text citations to ensure uniformity throughout the document, particularly in lines 354 and 358, aligning them with the citation style used in other paragraphs. For the third point, we have updated all online references by including the accession dates and direct links to the documents. As for the second point, since the journal follows the Vancouver style, which does not require DOIs, we have maintained the current citation format without adding DOI numbers. These revisions enhance the consistency and reliability of the references while adhering to the journal's guidelines.

**Reviewer 3：**

Reporting Guidelines

• The study aligns with the STROBE guidelines for observational studies, covering all key elements such as study design, methodology, results, and ethical considerations.

Supplementary materials

• The tables are not labelled as per PLOS ONE guidelines. Label them properly as per the journal guidelines.

• The subtitles in the one-way ANOVA (female) table should be all in English for easier understanding by the global audience.

• Define abbreviations as a footnote where applicable in the tables, e.g., LSD.

• Format the lines of the tables for uniformity and decency.

**The author’s answer:**

STROBE Guidelines Compliance:

The manuscript has been revised to fully align with the STROBE (Strengthening the Reporting of Observational Studies in Epidemiology) guidelines, ensuring comprehensive coverage of study design, methodology, results, and ethical considerations.

Table Formatting (PLOS ONE Guidelines):

All tables have been properly labeled in accordance with PLOS ONE requirements.

Subtitles in the one-way ANOVA (female) table have been standardized to English for clarity and global readability.

Abbreviations (e.g., LSD) are now clearly defined in footnotes where applicable.

Table lines and formatting have been adjusted for uniformity and professional presentation.

These revisions enhance the manuscript’s clarity, consistency, and adherence to reporting standards. We appreciate the opportunity to improve our work and hope the changes meet the reviewer’s expectations.

**Reviewer 4：**

Reviewer #4: The authors introduce a topic of interest to the international community on the trend of physical fitness and body weight throughout the COVID-19 pandemic in what is considered an active young adult population of university students.

The main concern of the manuscript is the missingness of data is not discussed. A study population of ~ 4,400 is described however data is only presented for ~10% of this population (~450) in the Results section without a proper discussion of loss of data/missingness in the Results or Discussion section. Secondly, the manuscript is exceedingly long, specifically the Results and Discussion sections and could use a major revision to streamline outcomes reported and interplay with current literature and findings in the Discussion. Please consider the suggested edits/comments detailed below in your revision:

**The author’s answer:**

We sincerely appreciate the reviewer’s insightful and constructive feedback. Regarding the concern about data missingness, we would like to clarify that the total study population (N ≈ 4,400) encompasses all enrolled participants. However, stratified analyses (e.g., by sex: males, n = 1,206; females, n = 3,207) were conducted to address specific research questions, ensuring methodological rigor and analytical precision.

Additionally, we have streamlined the Results and Discussion sections by removing redundant details, consolidating overlapping findings, and sharpening the focus on core outcomes and their interplay with existing literature. These revisions enhance clarity while maintaining the study’s substantive contributions.

**Reviewer 4：**

SPECIFIC RECOMMENDATIONS:

1- Suggest the start of a new paragraph with introduction of college lifestyle discussion [Ln 53].

2- Suggest condensing lines 56-71 with focus on the impact of COVID-19 on PA with reference to defined declines in the university population (or weight gain if PA data not specifically available for citation) .

**The author’s answer:**

We sincerely appreciate the reviewer's constructive suggestions. In response, we have revised the manuscript to: (1) introduce a clearer transition to college lifestyle discussions by separating the text into distinct paragraphs, and (2) condense the COVID-19 impact section (lines 56-71) to focus specifically on documented PA declines and weight gain among university students, with streamlined references to supporting literature [4-7]. These changes improve the flow while maintaining key evidence on pandemic-related behavioral shifts and their health implications.

**Reviewer 4：**

3- More clearly define the study population referenced in Line 86-87. How does this population compare or contrast to the current study population? Did the 3-year follow up occur post-university environment?

**The author’s answer:**

We appreciate the reviewer’s attention to methodological clarity. Regarding the study population referenced in Lines 86–87, we confirm that both the prior study and current research exclusively examined university students, with all follow-ups conducted within the campus environment. As no divergence existed in population characteristics (e.g., age, setting) or follow-up timing (both during university years), we omitted redundant comparisons in the Introduction to avoid unnecessary repetition.

**Reviewer 4：**

4- Lines 91-98 present similar context, consider merging.

5- Define body shape and how assessment will occur (Ln 93).

**The author’s answer:**

We sincerely appreciate the reviewer's constructive suggestions. In response to comments #4 and #5, we have: (1) merged the redundant context in Lines 91-98 to improve conciseness, (2) clearly defined "body shape" with specific assessment methods (anthropometric measurements including height, weight and BMI,), and (3) streamlined the paragraph while preserving all key study elements. The revised version maintains the study's scientific rigor while enhancing clarity and readability. These changes have improved the flow of the manuscript and better highlighted our unique longitudinal approach to examining pandemic impacts on student health. We believe these revisions have strengthened this section considerably.

**Reviewer 4：**

6-When defining the study population an age range/mean ± SD would be helpful (Ln 104) and ensure the proper word selection (e.g., boys/girls vs. men/women vs. male/female used interchangeably throughout) is appropriately used throughout.

**The author’s answer:**

We appreciate the reviewer’s valuable feedback. The study population consisted of 4,413 freshmen (1,206 male and 3,207 female students, mean age = 17.90 ± 0.90 years) recruited from a teacher training university in Hunan Province between September 5 and September 28, 2019, with a four-year follow-up throughout their university studies. We have ensured consistent use of male/female (instead of boys/girls or men/women) for uniformity across the manuscript. Thank you for your helpful suggestions.

**Reviewer 4：**

7- Under Methods section 2.2 please define the testing conditions. Was testing in the morning, environmental conditions, were groups held consistent to ensure competitive nature of participants were controlled for throughout the separate sessions? Were they similar across the 4 year testing period? Same test providers/raters?

**The author’s answer:**

In accordance with the suggestions made by the reviewers, relevant expressions have been added in section 2.2.

**Reviewer 4：**

8- Can you clarify if a time allotment was given for the pull-ups in Section 2.2.4?

**The author’s answer:**

In 2.2.4 it is clearly stated that there is no time requirement until the tester is exhausted.

**Reviewer 4：**

9-Consider combining Sections 2.2.5 and 2.2.6 since testing procedures were similar across both running sessions. Also, add the 800-m testing for women (Not currently mentioned).

**The author’s answer:**

We sincerely appreciate the reviewer's constructive suggestions. In response, we have combined Sections 2.2.5 (50-m run) and 2.2.6 (1,000-m run) into a single unified section titled "2.2.5 Running Tests (50-m, 800-m, and 1,000-m Runs)" to improve clarity and avoid redundancy, as the testing procedures were indeed similar. Additionally, we have incorporated the 800-m run for female participants, which was previously omitted, while maintaining the 1,000-m run for male students. The revised section now provides consistent descriptions of timing protocols, rounding rules, and attempt allowances across all running tests. These modifications have enhanced the manuscript's completeness and methodological rigor. Thank you for this valuable input.

**Reviewer 4：**

10- Were study participants limited to specific clothing during body weight assessment across the four timepoints? Consider adding this content to Line 172.

**The author’s answer:**

We have clarified that participants wore light indoor clothing during body weight measurements, in addition to removing their shoes, to ensure standardization across all four assessment timepoints.

**Reviewer 4：**

11- Were sex differences assessed for? Please elaborate why/why not? Would be of interest to the reader and all data appears available for analysis.

**The author’s answer:**

Indeed, sex differences were assessed in our analyses, as the data allowed for such comparisons between male and female participants. Given the biological and physiological distinctions between sexes in physical development and fitness parameters, these comparisons provide important insights into differential patterns among college students. The inclusion of sex-based analyses strengthens the study's relevance for developing tailored health promotion strategies.

**Reviewer 4：**

12-Typo Line 194, believe it should read “17≤YEAR≤19”

**The author’s answer:**

It has been modified.

**Reviewer 4：**

13-Why is the n so low in Table 2? Did you have missing data? If so, appears significant and needs to be discussed in Section 2.3 Statistical analysis section. Appears as if only 10% of total population data is presented in Table 2 & discussed.

**The author’s answer:**

14-Regarding the concern about data missingness, we would like to clarify that the total study population (N = 4,413) encompasses all enrolled participants. However, stratified analyses (e.g., by sex: males, n = 1,206; females, n = 3,207) were conducted to address specific research questions, ensuring methodological rigor and analytical precision.

**Reviewer 4：**

14- Define outcomes as sex or gender? Male/Female is considered sex, however gender is used (e.g., Ln 266, 365, 390).

**The author’s answer:**

The uniform use of “sex”.

**Reviewer 4：**

15-Results section is extremely long (6+ pages of text without tables and figures) and needs to be condensed. Much of the data presented in the tables is re-introduced in text. Consider a figure in place of Table 2 to show changes over time and condense outcome variables with similar trends from Tables to show similarities and streamline the discussion. Text used to describe outcomes is very similar across many sections of the Results (e.g., 266-269 and 291-294).

**The author’s answer:**

We appreciate the reviewer's constructive suggestions for improving the Results section. In response, we have: (1) consolidated outcome variables with similar patterns to highlight key findings more efficiently, and (4) eliminated repetitive text passages while maintaining all essential results. These modifications have streamlined the section while preserving the scientific rigor and completeness of our findings, making the results more accessible to readers without losing important information.

**Reviewer 4：**

16-Tables are difficult to read and decipher in the format currently inlayed within the text. Tables are also missing footnotes and units to define the variables.

17- Figure legends are missing to describe what population are displayed in Figure 1 and Figure 2.

**The author’s answer:**

Both the table and the picture have been modified and adjusted.

**Reviewer 4：**

18- Consider placement of Conclusion section prior to the Discussion section? Please check Journal layout and specifications.

**The author’s answer:**

The layout of the conclusion has been adjusted to place the conclusion after the discussion.

**Reviewer 4：**

19- Again, Discussion section is extremely lengthy (6+ pages) and needs to be condensed to summarize the outcomes and compare/contrast to literature in this area. Also, care to remove text and re-defining abbreviations already introduced again in the Discussion section.

**The author’s answer:**

We sincerely appreciate the reviewer's valuable suggestions for improving the Discussion section. In response, we have: (1) significantly condensed the section by removing redundant text and streamlining content, focusing on key findings and their implications; (2) strengthened comparisons with relevant literature to better contextualize our results; (3) eliminated re-definitions of abbreviations that were already introduced earlier in the manuscript; and (4) reorganized the discussion to maintain clarity while reducing length.

**Reviewer 4：**

20- How do the PA and overweight/obesity outcomes from your population compared to the University population without the influence of COVID-19? Are trends intensified for completely diverse?

**The author’s answer:**

Compared to pre-pandemic university populations, our COVID-19 cohort demonstrated intensified overweight/obesity trends, particularly among males, likely due to pandemic-related disruptions (e.g., prolonged campus lockdowns, reduced physical activity opportunities, and lifestyle changes). While pre-pandemic studies showed more stable trends, our findings reveal a distinct pattern: initial declines in overweight rates (2019–2021) followed by a sharp rebound in 2022, suggesting delayed or compounded effects of pandemic restrictions. These trends differ from non-COVID-19 studies, highlighting how public health crises may exacerbate weight-related risks in university settings. However, our female-majority sample may have partially moderated these effects, as females exhibited slower progression in obesity rates despite similar environmental pressures.

**Reviewer 4：**

21- The authors begin to compare how the study population compares with the adult or adolescent populations (post-COVID-19) previously published, realizing different environmental variables, etc. for consideration. However, data is not shown for level comparison, etc. Also, extraneous data provided on binge eating that is not covered in this study.

**The author’s answer:**

We appreciate the reviewer's insightful comment regarding comparative analyses with other populations. While our discussion highlights key contrasts between university students and adolescent/adult populations in post-COVID contexts , we acknowledge that formal statistical comparisons were not presented due to fundamental differences in study designs and environmental variables across these populations. We have removed any extraneous mentions of binge eating (which was not measured in our study) to maintain focus on our core physical fitness metrics. The revised discussion now more precisely frames these population comparisons as contextual observations rather than direct statistical contrasts, while still preserving their value for interpreting our unique findings within the broader literature on pandemic health impacts .

**Reviewer 4：**

22- Authors should be cautious with sweeping conclusions without data to support from dietary intake and body image outcomes assessed in Lines 368-370.

**The author’s answer:**

We thank the reviewer for this important observation. In response, we have removed the unsupported conclusions regarding dietary intake and body image outcomes from Lines 368-370, as these factors were not formally assessed in our study. The revised text now focuses strictly on interpreting the physical fitness and body composition data that were actually measured, ensuring all conclusions are fully evidence-based and aligned with our study design. We appreciate this opportunity to strengthen the manuscript's scientific rigor by eliminating any speculative claims beyond our dataset.

**Reviewer 4：**

23- Limitations section of the Discussion need to include the sample size used, missingness of data, etc.

**The author’s answer:**

Modifications were made to the limitations of the discussion section based on expert advice.

**Reviewer 5：**

Reviewer #5: Dear Author(s),

The current manuscript is not adding to the current literature. There are already studies as follows:

1-Ding, C., & Jiang, Y. (2020, December). The relationship between body mass index and physical fitness among Chinese university students: results of a longitudinal study. In Healthcare (Vol. 8, No. 4, p. 570).

2-Yu, H., An, S., Tao, Y., & Austin, L. (2022, September). Correlation and change in physical activity and physical fitness across four years of college students after one year of COVID-19 lockdown. In Healthcare (Vol. 10, No. 9, p. 1691).

3-Hao, Y., Lee, J., Wong, W. S. P., Wong, F. U. K., Hui, W. H. C., Leong, G. C. H., & Kong, Z. (2025). A longitudinal study to COVID-19 infection among university students: Physical fitness changes and psychological responses. Journal of Exercise Science & Fitness, 23(1), 7-13.

4-Martínez-de-Quel, Ó., Suárez-Iglesias, D., López-Flores, M., & Pérez, C. A. (2021). Physical activity, dietary habits and sleep quality before and during COVID-19 lockdown: A longitudinal study. Appetite, 158, 105019.

**The author’s answer:**

We sincerely appreciate the reviewer’s valuable feedback regarding the manuscript’s contribution to existing literature. We have carefully addressed this concern by: explicitly positioning our study’s novel aspects relative to the cited references (Ding & Jiang, 2020 [Ref 19]; Yu et al., 2022 [Ref 26]; Hao et al., 2025 [Ref 27]; Martínez-de-Quel et al., 2021 [Ref 8]), particularly our focus on sex-specific longitudinal trends in both physical fitness and obesity/overweight dynamics across all pandemic years (2019–2022)—a gap not fully covered by prior work.
