# Supplementary material for: A longitudinal study of four-year changes in physical fitness among university students before and after COVID-19: 2019–2022
Source: PLoS One. 2025 Oct 10;20(10):e0334088. doi: 10.1371/journal.pone.0334088 (PMC12513638; doi:10.1371/journal.pone.0334088)
Supplement: Attachment — Submitted filename: Response_to_Reviewers_auresp_2.docx [file pone.0334088.s005.docx]

Dear Editors and reviewers

On behalf of all the contributing authors, I would like to express our sincere appreciations of your letter and reviewers' constructive comments concerning our article entitled “A longitudinal study of four-year changes in physical fitness among university students before and after COVID-19: 2019-2022” (Manuscript No:PONE-D-25-29142R2). These comments are all valuable and helpful for improving our article. Based on the comments of the editor-in-chief and reviewers, we made extensive revisions to the manuscript.In this revised version. changes to our manuscript were all highlighted within the document by using red-colored text.Point-by point responses to the nice editor and four nice reviewers are listed below this letter.

**Point-by point responses**

**Reviewer 1：**

I would like to commend you on your careful and thorough revision of the manuscript “A longitudinal study of four-year changes in physical fitness among university students before and after COVID-19: 2019–2022.” The manuscript has improved substantially, both in scientific rigor and in clarity of presentation, and I appreciate the diligence with which you have addressed prior reviewer and editorial feedback.

Strengths of the Revised Manuscript:

The four-year longitudinal design is a notable strength, providing a unique opportunity to capture pre-pandemic baselines, acute pandemic effects, and post-pandemic adaptations. This adds considerable value to the literature, where shorter-term studies predominate.

Methodological transparency has been improved, with clear descriptions of inclusion/exclusion criteria, standardized testing protocols, assessor training, equipment calibration, and testing conditions. These additions enhance the reproducibility and reliability of the findings.

Tables and figures are now clearer and easier to interpret. The combination of innovative 3D scatterplots with boxplots provides both depth and accessibility in data visualization.

The revised Discussion is more structured, with explicit contextualization in relation to existing studies, as well as a balanced acknowledgment of limitations and future research directions.

Language and organization have been polished, making the manuscript more concise and accessible without loss of technical depth.

Conclusion:

Overall, this is a well-executed study that makes a meaningful contribution to understanding the long-term effects of the COVID-19 pandemic on university students’ physical fitness. The revisions have addressed previous concerns comprehensively, and the manuscript now meets the criteria for publication. I support its acceptance after minor editorial checks.

Congratulations on your careful work, and I look forward to seeing this valuable study published.

**The author’s answer:**

Thank you very much for your thoughtful and encouraging feedback on our revised manuscript, "A longitudinal study of four-year changes in physical fitness among university students before and after COVID-19: 2019–2022." We are truly grateful for your recognition of the efforts made to strengthen the manuscript's scientific rigor, clarity, and overall presentation.

We especially appreciate your positive comments regarding the longitudinal design, methodological transparency, improved data visualization, and the more structured and contextualized Discussion. It is very rewarding to know that the revisions have successfully addressed the previous concerns and enhanced the reproducibility, accessibility, and contribution of the study.

Your supportive conclusion and recommendation for acceptance are greatly encouraging. We are pleased to learn that the manuscript now meets the publication standards and will gladly cooperate with any minor editorial checks required.

Thank you once again for your time, insightful suggestions throughout the review process, and kind words.

**Reviewer 2：**

Thank you very much for your positive feedback and for confirming that our responses to your previous comments and suggestions are satisfactory. We greatly appreciate your time and insightful input throughout the review process, which has significantly improved the quality of our manuscript. We are pleased to know that you now consider the manuscript suitable for publication.

**Reviewer 4：**

1-Again, as asked in first review please revisit the use of gender vs. sex throughout. Currently both are used inter changeably [Ln 81,89,120,192,199,200]. Did you have transgender study participants? If so, consider use of gender otherwise correct use should be sex throughout.

**The author’s answer:**

Thank you for raising this point. We have carefully reviewed the use of “gender” and “sex” throughout the manuscript. As our study did not include any transgender participants, we have replaced all instances of “gender” in the specified lines (81, 89, 120, 192, 199, 200) and elsewhere in the text with the term “sex”. The manuscript has been updated accordingly.

**Reviewer 4：**

2-purposes? Or to better prevent and treat long-term health outcomes related to PA? [Ln 83-84; 90-91].

**The author’s answer:**

In the first modification, the phrase “long-term trends” was refined to “sustained trends” for terminological precision, while the scope of the “distinction” was expanded using “not only for... but also for...” to emphasize its critical role in shaping targeted strategies for managing PA-related health consequences. [Ln83-85]

In the second sentence, “for policymakers about” was strengthened to “to guide policymakers in”, enhancing the actionable nature of the evidence, and the vague “health impacts” was replaced with “preventing and mitigating long-term health outcomes associated with reduced physical activity”, thereby directly linking pandemic measures, PA reduction, and health outcomes as suggested by the reviewer. Both changes improve clarity, align with the commentary, and underscore the policy-responsive and intervention-oriented value of the study. [Ln91-93]

**Reviewer 4：**

3- Lines 101-107 read like a bulleted listing; needs to be integrated with the rest of the paragraph. In summary, ‘a convenience sample of available and interested incoming freshman class of 2019 admission with limited pre-existing health (e.g., ….) or physical conditions that may limit exercise participation’?

**The author’s answer:**

We thank the reviewer for this suggestion. The selection criteria description has been revised to improve integration within the paragraph, removing the bulleted-list format and presenting the information in a cohesive narrative form. The description now more clearly characterizes the sample as a convenience sample of available and interested freshmen with specific exclusions for health or physical conditions affecting exercise.[Ln104-111]

**Reviewer 4：**

4-It would be helpful for transparency if the authors included number of students/study participants remaining in study (data used) at each stage/year of the study in the Results similar to how you set up in Methods [Ln 113-116]. Is Table 2 correct? 1206 males and 3207 females remained in the study all 4 years? There was no drop out or data loss? Please make this clear in your Methods or Results section.

**The author’s answer:**

We thank the reviewer for raising this important point. We have now clarified the participant flow and retention details in the Methods section. Specifically, we have explicitly stated the initial number of participants, the number of dropouts over the study period, the reasons for attrition, and the final number of students (4413) with complete data for all four years. This revision enhances the transparency of our cohort's trajectory and confirms that the numbers presented in Table 2 reflect the participants with complete longitudinal data.[Ln120-125]

**Reviewer 4：**

5- Consider relocating details about testing administrators (e.g., physical education professors) from section 2.1 to Ln 125 or later for consistency. Were test administrators consistent throughout the study? This was asked in last review and not included in revision.

**The author’s answer:**

Thank you for this suggestion. We have relocated the details regarding the test administrators from section 2.1 to the methodological description of the testing procedures (as now reflected around line 125 in the revised manuscript) to improve the logical flow and consistency of information. We have also explicitly stated that the same team of trained professors conducted all assessments across all four years, directly addressing the previously raised question about the consistency of the testing personnel.

**Reviewer 4：**

6- Again, as asked in earlier review and authors did not respond or update Methods section 2.2 should define environmental conditions (e.g., room temperature and humidity ?) and testing groups participants were included in. These can greatly impact and bias outcomes (e.g., control for competitive nature of participants were controlled throughout sessions)

**The author’s answer:**

We thank the reviewer for this important feedback. We have now updated the Methods section (2.2) to include detailed information about the controlled environmental conditions (specifying room temperature and humidity ranges). These additions address potential confounding factors and enhance the reproducibility of our study protocol.[Ln138-139]

**Reviewer 4：**

7- Ln 141 is ‘blowing’ correct terminology to be used here?

**The author’s answer:**

Thank you for pointing this out. We have replaced the informal term “blowing” with the more precise and clinically appropriate terminology “exhalation maneuver” to accurately describe the spirometry procedure and enhance the scientific rigor of the manuscript.[Ln152]

**Reviewer 4：**

8- Ln 142 define ‘better’ here when decerning between two scores. Was there a pre-determined range?

**The author’s answer:**

We appreciate the reviewer’s comment for clarity. The term “better” has been replaced with the more objective and quantifiable term “higher” to describe the selection of scores, and we have explicitly noted that this was determined according to a pre-defined standardized scoring protocol, thereby eliminating any potential ambiguity.[Ln153-155]

**Reviewer 4：**

9- Ln 165 is ‘barbell’ correct terminology to be used here? Should text read ‘final number of pull-ups were recorded’?

**The author’s answer:**

We thank the reviewer for these precise suggestions. We have replaced the potentially ambiguous term “barbell” with the more accurate term “bar”, clarified the movement standard using the phrase "chins cleared the horizontal level of the bar," and rephrased the recording method to “the final number of correctly completed pull-ups was recorded” to enhance terminological and methodological precision.

**Reviewer 4：**

10- Define ‘coach’ in Ln 169, 175, (is this Physical Education Professor)? Also, can simplify text to “recorded time was rounded to nearest tenths of second”.

**The author’s answer:**

We thank the reviewer for these constructive suggestions. We have replaced the informal term “coach” with the more precise “physical education professor” throughout the text to maintain consistency with our methodology description, and have simplified the timing description to “recorded time was rounded to the nearest tenth of a second” for improved clarity and conciseness.[Ln183、189、191]

**Reviewer 4：**

11- Word missing in Ln 173 ‘was’

**The author’s answer:**

We thank the reviewer for this careful observation. The missing word “was” has been added to ensure grammatical correctness, and the sentence now reads: “Each participant completed two trials, with the better performance was recorded.”

**Reviewer 4：**

12- Is 1000m identical to 800m or 50m protocol? Please specify.

**The author’s answer:**

Thank you for raising this point for clarification. We have explicitly specified that the 1,000-meter run protocol was identical to the 800-m and 50-m runs in its starting procedure, timing method, and rounding rule to ensure consistency and clarity in the description of methodological protocols across all running tests.[Ln193-195]

**Reviewer 4：**

13- Ln 194-196 need to be added to Limitation section 4.4. in Discussion if not accounted for in the statistical analysis. Overlooking these confounding variables can greatly skew outcomes and interpretation.

**The author’s answer:**

We sincerely thank the reviewer for this insightful comment. We have now added the point regarding the potential confounding variables (age, baseline fitness, and socioeconomic status) to the Limitations section (Section 4.4) as suggested. This addition strengthens our discussion by acknowledging these important factors that could influence the interpretation of the results.[Ln]

**Reviewer 4：**

14- Ln 204 were individual data collectors consistent throughout the study? If not, please add interindividual variability of raters to your limitations section 4.4 in Discussion.

**The author’s answer:**

Thank you for this valuable comment. We agree that the consistency of data collectors is an important methodological consideration. In response to your feedback, we have revised the Limitations section (4.4) of the Discussion to explicitly acknowledge that multiple data collectors were involved due to the large scale and long duration of the study, and that this may have introduced inter-rater variability despite efforts to standardize protocols. The added sentence reads: "Furthermore, due to the large number of test items and participants, multiple data collectors were involved throughout the study period. Despite efforts to standardize protocols, this may have introduced inter-rater variability." We believe this addition strengthens the manuscript by providing a more comprehensive overview of the study's limitations.

**Reviewer 4：**

15- Tables 2 & 3 please add lines or shading for ease of reading, variable units of measure, and round values to significantly meaningful decimal place with consistency in Table 2.

**The author’s answer:**

Thank you for your constructive suggestions regarding the presentation of data in Tables 2 and 3. We have revised both tables accordingly by adding grid lines to enhance readability and to clearly delineate the data. Furthermore, we have ensured that all values in Table 2 are rounded to a consistent and statistically appropriate number of decimal places, and we have explicitly stated the units of measure for all relevant variables in the table headers.

**Reviewer 4：**

16- Trends are similar across sex and some performance outcomes reported in section 3.2.2. Suggest combining outcomes that are similar in trend into paragraphs when detailing Table 2 for simplicity and streamline Results section.

**The author’s answer:**

Thank you for your constructive suggestion. We have revised the Results section to combine outcomes with similar trends into more concise paragraphs, as recommended. The descriptions of vital capacity, long-jump, sit-reach, and endurance running tests have been grouped to improve clarity and flow, while all key statistical findings and temporal patterns have been retained.

**Reviewer 4：**

17- Again, as asked in original review figures are incomplete – axis labels and legends are missing to describe what population are displayed in Figure 1 and Figure 2.

**The author’s answer:**

We have now updated both Figure 1 and Figure 2 to include all necessary axis labels and legends to clearly describe the displayed populations and ensure the figures are complete and easily interpretable.

**Reviewer 4：**

18- Consider removing Figures 3 & 4 since Figures 1 &2 show same data.

**The author’s answer:**

Thank you for your suggestion. We agree that Figures 1 and 2 effectively present the same data as Figures 3 and 4. Therefore, we have removed Figures 3 and 4 from the manuscript to avoid redundancy and improve clarity.

**Reviewer 4：**

19- Reconsider use of ‘body shape’ as it misleads the reader (ex., header 4.1 and section). Authors did not measure any body lengths or circumferences. BMI is considered body size as is height and weight.

**The author’s answer:**

Thank you for this important feedback. We agree that the term "body shape" was misleading, as it implies measurements such as lengths or circumferences that were not conducted in our study. We have replaced "body shape" with the more accurate term "body size" throughout the manuscript, including in the header of section 4.1, to better reflect that our analysis was based on BMI, height, and weight.

**Reviewer 4：**

20- Ln 352-355 define the populations cited.

**The author’s answer:**

Thank you for this helpful suggestion. We have revised the manuscript to explicitly define the population as "the studied population of university students" in Lines 353-358. This change adds necessary clarity and ensures the cited group is precisely identified, as recommended.

**Reviewer 4：**

21- Consider a more appropriate reference for statement in Ln 357.

**The author’s answer:**

Thank you for the valuable suggestion. We agree that the reference provided is more appropriate and directly relevant to our statement regarding gender differences in overweight and obesity among college students. We have replaced the original citation with the suggested reference (Jiang et al., 2018)（22） in Line 359 of the revised manuscript.

**Reviewer 4：**

22- Ln 372-375 is the female to male ratio of students documented to be higher in the University selected in this study? This ration/proportion of population statistics should be stated.

**The author’s answer:**

Thank you for pointing this out. We have revised the manuscript to explicitly state the female to male ratio (approximately 3:1) of the student population at the selected university, as suggested. This provides clearer context for the sample composition and strengthens the discussion of potential selection effects. The modification can be found in Lines 374-379.

**Reviewer 4：**

23- Could differences across sex found in 50m run and sit-ups be linked to social norms and females becoming more familiarized with ‘working out’ in the University setting? There is work discussing this both in the civilian and military communities. Female may have a greater breath of improved performance because they start at a lower threshold than capable of achieving. Consider reviewing literature and citing in Discussion.

**The author’s answer:**

Thank you for this insightful suggestion. We have revised the Discussion to incorporate the point that sex-based differences in the 50-m run and sit-ups may be linked to social norms and females’ increased familiarity with structured exercise in the university setting. The following sentences have been added to the relevant paragraph:“These sex-specific improvements, particularly among females, may be partially attributed to social norms and increased familiarity with structured exercise in a university setting. As previous research in both civilian and military contexts has suggested, women often exhibit substantial gains in physical performance when provided with equal opportunities for training, potentially due to a lower initial baseline and thus greater room for improvement (Wills et al., 2023).”

**Reviewer 4：**

24- PA is defined in Ln 391, consider moving this earlier in the paper and use throughout.

**The author’s answer:**

Thank you for this valuable suggestion. We have revised the manuscript to define the abbreviation "PA" (for Physical Activity) upon its first occurrence in the text, which is now on line 49. This definition has been introduced earlier to ensure clarity for the reader. Furthermore, we have consistently used this abbreviation throughout the remainder of the paper.

**Reviewer 4：**

25- Section 4.4. needs to be added to include the significant disproportion of females in the study population skewing results, and items called out in Items # 6,13, and 14.

**The author’s answer:**

Thank you for these important observations. We have revised the Limitations section to incorporate the point regarding the potential bias introduced by the significant disproportion of females in our study population, as well as the additional methodological concerns raised in Items #6 , #13 , and #14. The final limitation now explicitly states that this gender imbalance may have skewed the results, and the broader methodological points have been integrated into the relevant existing limitations, strengthening our discussion of the study's constraints. The findings should therefore be interpreted with caution.

**Reviewer 4：**

26- Lastly, and most importantly in Ln 438 data missingness is presented as a limitation however nowhere in the manuscript is the attrition or missing data discussed. This needs to be addressed and authors need to be transparent with sample size across the four years.

**The author’s answer:**

Thank you for this critical feedback. We have now addressed the important issue of data missingness and attrition transparency by adding a dedicated subsection in the Methods that details the participant flow across the four-year study period. The manuscript now explicitly states: "A total of 4572 students participated in the baseline survey in 2019. Over the four-year period, 159 participants were lost to follow-up due to [briefly state reasons, e.g., voluntary withdrawal, transfer, or incomplete testing], resulting in a final analytical sample of 4413 students (1206 males and 3207 females) who completed all assessments across all four years, representing a retention rate of 96.5%."This addition ensures full transparency regarding sample size and attrition, directly addressing the limitation mentioned in Line 118-122.

**Reviewer 5：**

The paper is better and has improved. From abstract to references and tables and figures are revised accordingly.

Regards

**The author’s answer:**

Thank you for your positive feedback and for acknowledging the improvements made throughout the manuscript, including the abstract, references, tables, and figures. We greatly appreciate your time and constructive input, which has helped enhance the overall quality of the paper.
